# From family-supportive supervisor behavior to employee stewardship behavior: the serial mediating roles of work-family enrichment and individual thriving

**DOI:** 10.3389/fpsyg.2026.1878413

**Published:** 2026-10-23

**Authors:** ChuanHao Fan, Haodong Jiang, Han Wang, YunJie Xu

**Affiliations:** School of Business, Hohai University, Nanjing, China

**Keywords:** conservation of resources theory, employee stewardship behavior, family-supportive supervisor behavior, individual thriving, organizational psychology, work-family enrichment

## Abstract

**Introduction:**

Under rapid digitalization and increasingly blurred work-family boundaries, organizations face the challenge of protecting employees’ psychological resources while fostering stewardship toward long-term organizational interests. Although family-supportive supervisor behavior provides an important contextual resource for employees, the resource-based process through which such family-supportive supervision translates into Employee Stewardship Behavior remains insufficiently explained.

**Methods:**

Drawing on Conservation of Resources Theory, this study develops a serial mediation model to examine how Family-Supportive Supervisor Behavior is associated with Employee Stewardship Behavior through Work-Family Enrichment and Individual Thriving. Matched data were collected from 506 employees and their immediate supervisors in China using a three-wave, multi-source survey design.

**Results:**

The results show that Family-Supportive Supervisor Behavior is positively associated with Employee Stewardship Behavior. Moreover, Individual Thriving independently mediates this relationship, whereas Work-Family Enrichment contributes to Employee Stewardship Behavior primarily through its serial pathway via Individual Thriving. The downstream pathways linking Work-Family Enrichment and Individual Thriving to Employee Stewardship Behavior appear relatively stable across employee subgroups.

**Conclusion:**

This study advances organizational psychology research by clarifying how supervisory practices that support work-life balance are related to cross-domain enrichment, psychological resources and future-oriented organizational responsibility.

## 1 Introduction

In contemporary organizations, employees are increasingly required to manage multiple role demands across work and family domains while maintaining sufficient psychological resources for sustained work engagement and long-term organizational contribution. Rapid digitalization, intensified work demands, and increasingly blurred work-family boundaries have made this challenge more salient. In high-velocity work environments, work demands increasingly encroach upon family life (Rothbard and Edwards, 2003) and blur work-family boundaries (Shen et al., 2022; Wang et al., 2018). Such boundary blurring may increase work–family conflict, performance decline and burnout, while also engendering broader family-related consequences such as parental burnout (Li et al., 2024) and weakened fertility intentions (Zhang and Song, 2024). Therefore, understanding how organizations can help employees protect and transform psychological resources under growing work-family pressures is an important issue in organizational psychology.

Beyond traditional dual-earner parenting, contemporary family-role pressures also take forms not fully captured by marital or parental status alone. Delayed marriage, non-marriage, and DINK (double income, no kids) patterns are increasingly visible, and many younger employees still invest substantial time and emotional resources in origin-family obligations, anticipated eldercare, intimate partnerships, and dual-career coordination. In this broader sense, “family” responsibilities are not limited to married employees with children. Clarifying how supervisory support at the work-family interface relates to employees’ long-term organizational responsibility therefore remains relevant even in relatively young samples with a sizable unmarried share.

Supervisors, as key providers and allocators of organizational resources, play a pivotal role in this process. Family-Supportive Supervisor Behavior (FSSB) refers to supervisors’ behaviors that support employees in fulfilling family responsibilities, including emotional support, instrumental support, role modeling, and creative work-family management (Hammer et al., 2006). Unlike general supervisory support, FSSB directly targets the work-family interface, where employees often encounter competing demands for time, energy, and emotional resources. By providing family-specific support, supervisors may help employees reduce resource depletion, coordinate work and family roles, and develop more positive experiences across domains. Consequently, examining how FSSB helps employees maintain and transform psychological resources across work and family domains is important for understanding the organizational conditions that support employee functioning under work-family pressures.

Prior research has shown that FSSB can improve work-family experiences and work-related outcomes (Jiang et al., 2015; Shen et al., 2022; Wu et al., 2020), suggesting that it may serve as an important contextual resource in the work-family interface. However, the significance of FSSB for organizational management may extend beyond reducing immediate work–family conflict. A key question is whether such support can also encourage employees to assume long-term responsibility for organizational interests. Employee Stewardship Behavior (ESB) provides a meaningful behavioral outcome for examining this issue. ESB refers to discretionary behavior in which individuals prioritize the organization’s long-term interests, even at the expense of short-term personal gains, when making decisions and taking action (Hernandez, 2012). Prior research has shown that ESB is associated with individual creativity (Kuppelwieser, 2011). It is also associated with employees’ willingness to internalize organizational interests and act beyond narrow self-interest under uncertainty (Hernandez, 2008; Wang and Zhao, 2020). Compared with commonly examined pro-organizational behaviors, such as organizational citizenship behavior and voice behavior, ESB places stronger emphasis on long-term orientation, responsibility, and potential self-sacrifice (Hernandez, 2012).

Existing research has provided initial evidence that FSSB can promote ESB both directly and indirectly through perceived insider status (Wang and Zhao, 2020). This finding establishes FSSB as a relevant antecedent of ESB, but the underlying explanation has largely followed a relational and identity-based logic. However, because FSSB operates at the work-family interface, its contribution to ESB may depend not only on whether employees feel recognized by the organization, but also on whether supervisory support helps employees sustain and transform resources across life domains. Conservation of Resources Theory (COR) provides an appropriate theoretical lens for specifying this resource-based explanation. COR suggests that individuals strive to protect, acquire, and invest resources, and that behavioral choices depend on whether resources are threatened, depleted, or sufficiently available (Hobfoll, 1989, 2001). Accordingly, FSSB may be understood as a supervisory resource that supports ESB by reducing cross-domain resource depletion, facilitating resource gains, and strengthening employees’ capacity and willingness to reinvest resources in long-term organizational goals, rather than only through relational membership perceptions.

Because FSSB directly concerns the coordination of work and family roles, its resource-based effect should be reflected in whether employees can transform supervisory support into gains across domains. Work-Family Enrichment (WFE) provides a suitable construct for explaining this cross-domain resource conversion. WFE refers to the process through which resources and positive experiences acquired in one domain improve functioning in another domain (Greenhaus and Powell, 2006). Prior research suggests that FSSB is positively associated with WFE, indicating that FSSB can help employees transform supervisory resources into positive work-family experiences (Shen et al., 2022; Wu et al., 2020). However, WFE has mainly been examined as a mediating mechanism for explaining relatively proximal work outcomes (Deng et al., 2024), whereas its role in supporting long-term responsibility-oriented behavior has received less attention. From the perspective of COR, such cross-domain resource gains may enhance employees’ energy, flexibility, and resource security, making them more capable of investing resources in long-term organizational interests. Accordingly, WFE offers a resource-conversion explanation for how FSSB may contribute to ESB.

Beyond cross-domain resource conversion, the resource implication of FSSB can also be understood in relation to evidence that organizational support fosters a psychological state that connects supportive resources with positive work outcomes (Abid et al., 2015). Individual Thriving (THR) refers to a positive psychological state encompassing both vitality and learning (Porath et al., 2012). These two dimensions are relevant to ESB because it requires employees to maintain sustained energy, assume responsibility, and tolerate potential short-term personal costs in pursuit of long-term organizational interests. THR is also closely connected with WFE. WFE can promote both thriving at work (Russo et al., 2018) and thriving at family while family-work enrichment promotes thriving at work (Han et al., 2020). Related research has also shown that FSSB, as a work-related resource, can promote employee thriving through WFE (Wang et al., 2022). These findings suggest that cross-domain resource gains may provide an important developmental basis for employees’ vitality and learning. Accordingly, THR offers a psychological resource explanation for how FSSB may contribute to ESB, while also helping clarify how WFE may be transformed into long-term stewardship behavior.

Taken together, building on prior research linking FSSB to ESB through a proactive motivation perspective (Wang and Zhao, 2020), this study develops a COR-based serial mediation model to explain how family-supportive supervisory resources are converted and internalized into long-term stewardship behavior. Specifically, WFE is positioned as a cross-domain resource enrichment process through which FSSB expands employees’ available resources, whereas THR is positioned as a psychological resource state through which employees develop sustained vitality and learning for ESB. The study explains how FSSB, as a supervisor-provided resource, may contribute to long-term organizational responsibility by fostering work-family resource enrichment and employee psychological thriving. The findings contribute to research on long-term pro-organizational behavior and organizational psychology by clarifying the psychological resource mechanisms through which supervisory practices that support work-family balance foster employee development and organizational responsibility.

## 2 Theoretical foundations and research hypotheses

### 2.1 Theoretical foundations

Conservation of Resources Theory, proposed by Hobfoll (1989, 2001), provides a theoretical framework for understanding how individuals preserve, acquire, and invest resources. COR suggests that individuals possess a fundamental motivation to preserve valued resources, prevent resource loss, and continuously obtain new resources. When existing resources are depleted, threatened, or fail to generate expected returns, individuals are more likely to experience stress and adopt defensive behavioral strategies. In contrast, when resource availability is perceived as sufficient and stable, individuals are more likely to invest resources in activities that may generate future returns.

A central proposition of COR is that resources are transferable and cumulative rather than isolated. Resources acquired in one domain may facilitate functioning in another domain through resource transfer, compensation, and conversion mechanisms. Building on this logic, COR further proposes resource loss spirals and resource gain spirals (Hobfoll, 2002; Hobfoll et al., 2018). Initial resource loss may trigger further depletion, whereas initial resource gain may increase the likelihood of acquiring additional resources. This logic is particularly relevant to the work-family interface, where employees continuously allocate time, energy, attention, and emotional resources across work and family roles. Under intensified work-family pressures, employees may shift toward resource conservation and short-term cost control. Conversely, when resources can circulate and accumulate across domains, employees may develop a stronger basis for psychological functioning and long-term behavioral investment.

In addition, COR emphasizes the interaction between individual resources and organizational contextual resources. Organizational contexts and management practices may either intensify resource depletion or provide external resources that help employees maintain adaptive psychological functioning. From an organizational psychology perspective, this means that people-centered management practices are important not only because they reduce immediate strain, but also because they shape employees’ resource availability, resource conversion, and resource investment. In this sense, FSSB can be understood as a supervisory resource that provides emotional, instrumental, and work-family management support. Such support may help employees reduce resource depletion and create conditions for transforming resources across work and family domains.

Within this COR-based framework, WFE, THR, and ESB represent different stages of the resource process. Although both constructs are resource-related and may correlate empirically, WFE and THR occupy different theoretical positions. WFE (Greenhaus and Powell, 2006) is a cross-role resource-gain process: resources and positive experiences acquired in one domain improve functioning in another. THR (Porath et al., 2012; extended here to work and family) is a psychological resource state combining vitality and learning, reflecting whether resources have been internalized into relatively stable psychological functioning. Under COR, WFE describes how resources flow and enrich across roles; THR describes whether the individual experiences the energy and developmental momentum needed for long-term investment. WFE reflects the cross-domain resource enrichment process through which employees transform support and positive experiences into gains across work and family roles. THR reflects a psychological resource state in which employees experience vitality and learning, indicating that external and cross-domain resources have been internalized into a more stable form of psychological functioning. ESB, in turn, requires employees to prioritize long-term organizational interests and tolerate potential short-term personal costs. From the perspective of COR, such behavior can be interpreted as a form of long-term resource investment that becomes more likely when employees possess sufficient resource reserves and stable expectations regarding future resource availability.

The selection of COR as the primary theoretical lens, and of WFE and THR as the two mediators, requires justification relative to alternative frameworks. Social exchange theory, leader–member exchange, and job demands–resources theory can also illuminate supportive supervision, but each primarily emphasizes reciprocal obligation, relationship quality, or demand–resource balance rather than the full resource-protection, conversion, internalization, and investment sequence proposed here. Work-Home Resources theory is closely affiliated with COR and complements rather than substitutes for the resource-sequence logic; organizational identification and stewardship theory primarily operate through relational or identity-based mechanisms, which this study incorporates but does not rely upon as the primary explanatory pathway. COR is particularly suited to the present research question because FSSB is theorized as a contextual resource that protects employees from cross-domain depletion and enables subsequent resource investment. Within this framework, WFE captures the conversion of supervisory support into gains that transfer across work and family roles, whereas THR captures whether those gains are internalized as vitality and learning—the psychological conditions for sustained, future-oriented responsibility. Together, WFE and THR thus specify a resource pathway from family-supportive supervision to stewardship behavior that extends beyond a relational or identity-based explanation alone. Drawing on the above theoretical logic, COR provides an integrated framework for explaining how FSSB may promote ESB through resource protection, cross-domain resource conversion, psychological internalization, and long-term resource investment. Based on COR, this study further develops research hypotheses to examine the mechanisms among relevant variables.

### 2.2 Family-supportive supervisor behavior and employee stewardship behavior

Prior research has shown that FSSB can promote ESB through a proactive motivation process, particularly by enhancing employees’ perceived insider status (Wang and Zhao, 2020). Building on this evidence, this study further interprets the FSSB-ESB relationship from a COR perspective. This study emphasizes how FSSB functions as a supervisory resource that protects employees’ resources and enables long-term resource investment rather than focusing only on relational membership perceptions.

In contexts where work and family role demands coexist, employees must continuously allocate time, energy, and emotional resources across domains. When these demands intensify, employees are more likely to experience resource strain and focus on immediate task completion and short-term cost control rather than long-term organizational goals. From the perspective of COR, resource strain may increase employees’ sensitivity to potential resource loss and reduce their willingness to engage in behaviors involving uncertainty and short-term personal costs (Hobfoll, 1989, 2001; Hobfoll et al., 2018).

FSSB can weaken this resource-conservation tendency by providing employees with supervisory resources for managing work and family demands. Through emotional support, instrumental assistance, role modeling, and creative work-family management, FSSB helps employees reduce resource depletion during role transitions and strengthens their expectations regarding resource stability and recoverability (Hammer et al., 2006). As employees perceive that their resources are more protected and replenishable, they are less likely to remain in a defensive resource state and more likely to redirect attention from short-term cost control toward longer-term goals.

This resource-based logic is directly relevant to ESB. ESB reflects employees’ willingness to prioritize organizational long-term interests, even at the expense of short-term personal gains, when making decisions and taking action (Hernandez, 2008, 2012). ESB therefore differs from traditional organizational citizenship behavior in both mindset and time horizon. Whereas OCB typically involves general helping, courtesy, or conscientiousness directed at colleagues or the organization in the short term, ESB reflects an owner-like stewardship mindset centered on protecting the organization’s long-term interests and accepting potential short-term personal costs under uncertainty. Although ESB and OCB may correlate empirically, the present study treats long-term stewardship orientation rather than general extra-role helping as the focal outcome. From a COR perspective, ESB can be interpreted as a form of long-term resource investment in which employees devote time, energy, and responsibility to organizational interests under uncertainty. When FSSB helps employees maintain sufficient resource reserves and perceive lower risk of resource loss, employees become more capable of tolerating short-term costs and assuming responsibility for future-oriented organizational interests. Therefore, FSSB, as a supervisory resource, is expected to promote ESB by enabling employees to shift from resource conservation toward long-term resource investment. Based on this, the study proposes the following hypothesis:

*Hypothesis 1*: Family-Supportive Supervisor Behavior has a positive effect on Employee Stewardship Behavior.

### 2.3 Mediating role of work-family enrichment

From the perspective of COR, WFE represents a cross-domain resource enrichment process. Compared with the reduction of work–family conflict, WFE more directly captures the resource gain side of COR because it emphasizes how time, energy, skills, positive emotions, and supportive experiences can be transferred and amplified across work and family roles. Therefore, WFE is significant for explaining how family-supportive supervisory resources are transformed into broader resource gains.

FSSB provides an important contextual condition for the formation of WFE. Through emotional support, instrumental assistance, role modeling, and creative work-family management, supervisors can help employees coordinate work and family demands and reduce unnecessary resource depletion during role transitions (Hammer et al., 2006). Previous research suggests that FSSB can both alleviate work–family conflict and promote WFE by providing emotional and instrumental resources (Jiang et al., 2015; Wang et al., 2022). Related studies have also shown that FSSB is positively associated with WFE based on social exchange theory and work-home resource model, indicating that family-supportive supervision can help employees convert supervisory support into positive work-family experiences (Shen et al., 2022; Wu et al., 2020). When employees receive family-supportive resources from supervisors, they are more likely to experience resource availability, flexibility, and emotional stability, which enables resources generated in the work domain to benefit family functioning and resources generated in the family domain to reinforce work functioning, thereby forming a positive cycle of interaction and resource circulation between work and family. Based on this, the study proposes the following hypothesis:

*Hypothesis 2a*: Family-Supportive Supervisor Behavior has a positive effect on Work-Family Enrichment.

WFE may further contribute to ESB because cross-domain resource gains can increase employees’ capacity for long-term resource investment. Existing research has mainly examined WFE as a mediating mechanism for explaining relatively proximal outcomes, such as work-family balance, work engagement, performance, and other immediate work-related outcomes (Deng et al., 2024; Ye et al., 2024; Zhao et al., 2024). However, from the perspective of COR, WFE is not limited to short-term role functioning. When employees experience enrichment between work and family domains, their overall resource pool is expanded through repeated resource transfer and accumulation. This resource gain state may reduce employees’ sensitivity to potential resource loss and strengthen their perceived resource security. As a result, employees become more capable of allocating time, energy, and responsibility to organizational goals that extend beyond immediate personal benefits. Since ESB requires employees to prioritize long-term organizational interests and tolerate potential short-term personal costs, WFE can provide an important resource basis for such behavior. Based on this, the study proposes the following hypothesis:

*Hypothesis 2b*: Work-Family Enrichment has a positive effect on Employee Stewardship Behavior.

Overall, FSSB provides employees with supervisory resources that support work-family coordination and promotes resource conversion and reinforcement between work and family, thereby facilitating entry into a resource gain spiral. In this process, WFE explains how FSSB, as a supervisory resource, is converted into cross-domain resource gains that support ESB, making employees more likely to shift from behavior patterns oriented toward short-term resource conservation to responsibility-taking oriented toward long-term resource returns. Therefore, WFE plays a critical mediating role in the relationship between FSSB and ESB. This reasoning leads to the following hypothesis:

*Hypothesis 2c*: Work-Family Enrichment mediates the relationship between Family-Supportive Supervisor Behavior and Employee Stewardship Behavior.

### 2.4 Mediating role of individual thriving

THR can be understood as an internal psychological resource state that reflects whether employees possess sufficient energy and developmental momentum to cope with demands and pursue future-oriented goals. THR has often been associated with positive work outcomes such as innovative behavior and reduced turnover intention (Abid et al., 2015). Unlike temporary positive affect, THR indicates a more sustainable form of psychological functioning because vitality provides the energy basis for continued engagement, while learning provides the cognitive and developmental basis for growth-oriented action.

FSSB provides an important contextual resource for the development of THR. Supervisors can help employees reduce resource depletion caused by competing work and family demands, which may increase employees’ perceived resource availability, psychological availability, and sense of control over work-family role coordination. When employees perceive that supervisory resources are available and usable, they are more likely to maintain vitality and allocate attention to learning and development rather than remaining in a defensive state of resource conservation. Prior research has shown that perceived organizational support can foster employee thriving and connect supportive resources with positive work outcomes (Abid et al., 2015). Relevant studies have also suggested that FSSB is associated with thriving-related outcomes (Qin et al., 2022; Russo et al., 2018). Based on this, the study proposes the following hypothesis:

*Hypothesis 3a*: Family-Supportive Supervisor Behavior has a positive effect on Individual Thriving.

THR may further promote ESB because vitality and learning provide psychological conditions for long-term resource investment. ESB requires employees to prioritize organizational long-term interests, assume responsibility under uncertainty, and tolerate potential short-term personal costs. Employees experiencing THR possess greater energy for sustained effort and stronger developmental orientation for handling uncertain or demanding situations. From the perspective of COR, such employees are less likely to focus solely on immediate resource preservation and more likely to invest resources in actions with long-term value. Existing studies have shown that THR is associated with positive employee outcomes, including innovative work behavior, performance, and reduced turnover intention (Abid et al., 2015; Gerbasi et al., 2015). These findings suggest that thriving can support employees’ sustained contribution beyond immediate task fulfillment. Accordingly, employees with higher THR are more likely to engage in ESB by devoting energy, learning capacity, and responsibility to long-term organizational interests. Based on this, the study proposes the following hypothesis:

*Hypothesis 3b*: Individual Thriving has a positive effect on Employee Stewardship Behavior.

Taken together, FSSB may foster THR by providing supervisory resources that reduce depletion and support vitality and learning, while THR may further enhance employees’ capacity and willingness to engage in long-term stewardship behavior. In this process, supportive resources obtained from the organizational context are internalized into psychological resources, enabling employees to move from resource conservation and stress coping toward resource accumulation and investment orientation. Therefore, THR explains how supervisory resources are transformed into psychological resources that support ESB and is expected to mediate the relationship between FSSB and ESB. We therefore hypothesize the following:

*Hypothesis 3c*: Individual Thriving mediates the relationship between Family-Supportive Supervisor Behavior and Employee Stewardship Behavior.

### 2.5 Serial mediation of work-family enrichment and individual thriving

From the perspective of COR, cross-domain resource gains may further support employees’ psychological resource development. WFE theory indicates that resources, positive experiences, and role-related capabilities can be transferred and amplified between work and family domains. When employees experience WFE, they are less likely to perceive work and family roles as competing demands and more likely to experience them as mutually reinforcing resource domains. This resource enrichment process can provide employees with greater energy, positive emotions, and developmental opportunities, thereby supporting the formation of THR. Prior research from a resource enrichment perspective has shown that work-family enrichment contributes to thriving in the family domain, while family-work enrichment contributes to thriving in the work domain (Han et al., 2020). Related studies have also indicated that WFE plays an important role in linking work-related resources, including FSSB, to employee thriving (Qin et al., 2022; Russo et al., 2018; Wang et al., 2022). Thus, WFE may provide the developmental resource basis through which employees experience greater vitality and learning. In the proposed resource sequence, WFE and THR occupy different theoretical positions. WFE is a cross-domain resource-conversion process through which resources are transferred and amplified across work and family roles, whereas THR is a psychological resource state characterized by vitality and learning. For the present research question, WFE is therefore positioned as the more proximal process through which family-supportive supervisory resources become cross-domain gains, and THR as the subsequent state in which these gains are internalized. Although reverse or reciprocal relationships between WFE and THR are conceivable, the proposed WFE → THR order is theoretically preferred rather than established by measurement timing alone. Based on this, the study proposes the following hypothesis:

*Hypothesis 4a*: Work-Family Enrichment has a positive effect on Individual Thriving.

The joint roles of WFE and THR further suggest a serial resource process linking FSSB to ESB. FSSB, as a supervisory resource, helps employees coordinate work and family demands and transform supervisory support into cross-domain resource gains. These resource gains, reflected in WFE, expand employees’ available resources and create conditions for psychological development. As employees internalize these cross-domain gains into THR, they become more likely to possess the sustained vitality and learning orientation needed for long-term resource investment. Since ESB requires employees to prioritize organizational long-term interests and tolerate potential short-term personal costs, THR represents the psychological resource state through which WFE can be transformed into stewardship behavior. Therefore, FSSB may be associated with ESB through a sequential process of work-family resource enrichment and psychological thriving. The multi-wave design provides temporal separation among the focal constructs, but it does not by itself establish causal ordering; reverse or reciprocal processes should be examined in future research. Based on this, the study proposes the following hypothesis:

*Hypothesis 4b*: Work-Family Enrichment and Individual Thriving exert a serial mediating effect in the relationship between Family-Supportive Supervisor Behavior and Employee Stewardship Behavior.

In summary, this study proposes the serial mediation theoretical model illustrated in Figure 1.

**Figure 1:**
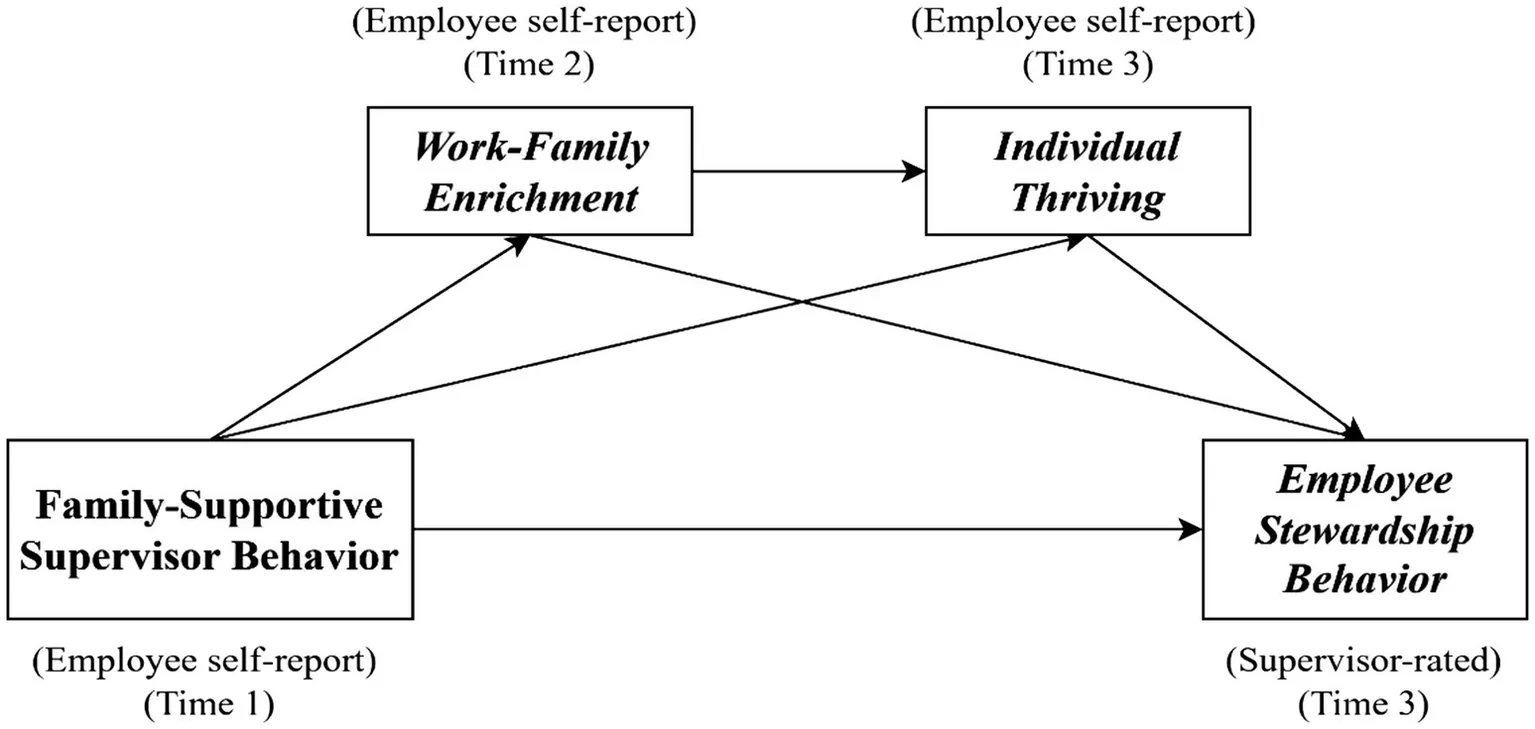
Research model.

## 3 Materials and methods

### 3.1 Sample and procedures

Following the procedural control recommendations proposed by Podsakoff et al. (2003), a multi-wave and multi-source questionnaire design was adopted, and data were collected through employee-supervisor matching to reduce common method bias at the procedural level. The research sample consisted of full-time employees and their direct supervisors from different regions of China. Participation was voluntary, and informed consent was obtained before survey participation. Participants were drawn from different organizations and regions in China to improve sample heterogeneity. Data were collected at three time points, each separated by two-week intervals. The two-week lags were selected to provide temporal separation for key constructs while maintaining feasibility and minimizing attrition in a three-wave matched design. To match responses across time points and data sources, the last four digits of participants’ mobile phone numbers were used as a one-time matching identifier, after providing full disclosure of the study purpose and guaranteeing anonymity and confidentiality. These digits were used solely for matching the three waves and the supervisor ratings, were not combined with any other personal identifiers, and are not part of the analytical dataset. The specific data collection procedures were as follows. At Phase 1 (Time 1), employee questionnaires were distributed to collect demographic information and self-reported FSSB data, with 643 questionnaires distributed. At Phase 2 (Time 2), conducted 2 weeks after Time 1, the same group of employees completed follow-up surveys measuring WFE. At Phase 3 (Time 3), conducted 2 weeks after Time 2, employees reported THR, and their direct supervisors evaluated ESB. After data collection was completed, questionnaires showing patterned responses, excessively short completion times, or unsuccessful matching were excluded. Ultimately, 506 valid employee-supervisor matched datasets were obtained, resulting in a valid matching rate of 78.69%. Because wave-specific retention and exclusion counts for Time 2 and Time 3 were not retained in the dataset, attrition statistics for the intermediate waves cannot be reported; the final analytical sample (*N* = 506) reflects all exclusion criteria (patterned responses, excessively short completion times, and unsuccessful matching) applied after data collection. Because the exclusion screening was performed after data collection without retaining per-reason counts, the number of cases excluded for each specific reason (patterned responses, excessively short completion times, and unsuccessful matching) cannot be reported either. The recruitment strategy combined online and offline distribution across multiple organizations and regions in China without restricting industry or firm type; information on the number, industry, and size of participating organizations was not collected. These records were not obtained, which limits the characterization of the sampling frame and the assessment of generalizability.

The demographic characteristics of the sample (*N* = 506) were as follows. Male participants accounted for 40.12% and female participants accounted for 59.88%. Regarding age distribution, the five-category distribution was: 25 years or below, 145 (28.66%); 26–35 years, 219 (43.28%); 36–45 years, 117 (23.12%); 46–55 years, 20 (3.95%); and 56 years or above, 5 (0.99%). Thus, participants aged 35 or below accounted for 364 (71.94%), and those aged above 35 accounted for 142 (28.06%). Regarding education level, participants with high school (vocational secondary school) or below, junior college, bachelor’s degree, master’s degree, and doctoral degree accounted for 3.16, 12.45, 60.47, 21.34, and 2.57%, respectively. Regarding marital status, unmarried and married participants accounted for 52.96 and 47.04%, respectively. Regarding job level, 46.25% were frontline employees and 53.75% were frontline managers (including team leaders and supervisors). Regarding supervisor gender characteristics, 60.47% of supervisors were male and 39.53% were female. Regarding supervisor age distribution, 3.56% were aged 25 or below, 22.92% were aged 26–35, 52.17% were aged 36–45, 18.38% were aged 46–55, and 2.96% were aged 56 or above.

### 3.2 Measurement

All key variables were measured using established and validated scales. All items were measured using five-point Likert scales. Scale translation followed a translation and back-translation procedure to ensure semantic equivalence, and empirical validation was conducted based on survey data.

FSSB was measured using the 14-item multidimensional scale developed by Hammer et al. (2006), including Emotional Support (4 items), Instrumental Support (3 items), Role Modeling (3 items), and Creative Work-Family Management (4 items). The Cronbach’s *α* coefficient was 0.935.

WFE was measured using a 14-item scale developed and adapted by Chinese scholars. Based on Carlson et al. (2006) and semi-structured interviews, Tang developed a Work-Family Enrichment scale suitable for the Chinese context (Tang et al., 2009), including 7 items measuring work-to-family enrichment and 7 items measuring family-to-work enrichment. The Cronbach’s *α* coefficient was 0.922.

Following the approach adopted in prior studies (Chen and Fellenz, 2020; Lin et al., 2022; Yang and Lin, 2017), this study measured THR by extending the Work Thriving scale developed by Porath et al. (2012) to both work and family domains. The scale consisted of 20 items across four dimensions: work vitality, work learning, family vitality, and family learning, thereby capturing employees’ thriving experiences in both work and family contexts. Sample items include “I feel alive and vital at work” (work vitality) and “I continue to learn more as time goes by in my family life” (family learning; wording adapted to domain). Confirmatory factor analyses supported the four-construct measurement model (FSSB, WFE, THR, ESB), and the hypothesized model demonstrated acceptable fit and superior performance relative to alternative models. THR was operationalized in two ways across the analyses, each serving a distinct purpose: in the CFA used to validate the measurement model and assess construct distinctiveness (Table 1), THR was specified as a single first-order factor indicated by its retained items, alongside first-order factors for FSSB, WFE, and ESB; in the latent SEM used to test the hypothesized model, THR was specified as a higher-order construct: the second-order THR factor was indicated by two first-order domain factors, work thriving (work vitality and work learning items, CA1–CA10) and family thriving (family vitality and family learning items, CB1, CB3–CB6, CB8, CB9). The higher-order factor was identified by fixing the loading of work thriving to 1 (marker-variable identification) and freely estimating the loading of family thriving and the second-order variance; the standardized loadings of work thriving (0.939) and family thriving (0.863) on the higher-order factor were substantial. The Cronbach’s *α* coefficient was 0.937. FSSB, WFE, and ESB were specified identically in the CFA and the SEM: single overall latent factors indicated by their retained items (FSSB: 14 items; WFE: 10 retained items; ESB: 3 items).

**Table 1:** Comparison of model fit among the four-factor model and competing models.

Model	*χ*^2^/df	RMSEA	TLI	CFI	IFI
Four-factor benchmark model (FSSB, WFE, THR, ESB)	2.800	0.060	0.874	0.880	0.881
Three-factor model (FSSB + WFE, THR, ESB)	4.430	0.082	0.759	0.771	0.772
Two-factor model (FSSB + WFE + THR, ESB)	5.467	0.094	0.686	0.701	0.702
Single-factor model (FSSB + WFE + THR + ESB)	6.002	0.099	0.649	0.665	0.666

*N* = 506. Estimated in R (lavaan) with maximum likelihood; the four-factor benchmark model includes the 44 retained items, and competing models are constructed by merging factors; all models are specified without error covariances.

ESB was measured using the three-item unidimensional Employee Stewardship Behavior scale developed by Davis et al. (2010). Although the ESB scale was originally developed in a family business context, the three items capture an individual-level stewardship orientation (i.e., prioritizing the organization’s long-term interests and accepting potential short-term personal costs), which is conceptually applicable to employees in general organizational settings. Moreover, this operationalization has been adopted in subsequent studies across broader organizational contexts beyond family firms (Wang and Zhao, 2020; Yan et al., 2020). During data collection, employees’ direct supervisors evaluated ESB. The Cronbach’s *α* coefficient was 0.893.

## 4 Analysis and results

### 4.1 Data analysis

#### 4.1.1 Reliability and validity

Reliability analysis indicated that Cronbach’s *α* coefficients for all scales were at satisfactory levels (FSSB = 0.935; WFE = 0.922; THR = 0.937; ESB = 0.893), indicating good internal consistency.

Confirmatory factor analysis (CFA) was conducted to evaluate the measurement model. Following the recommendations of Hair et al., items with relatively low standardized factor loadings were removed to ensure measurement quality and convergent validity (Hair et al., 2014). During the CFA assessment, seven items from the original 51-item pool were excluded: BB4–BB7 (family-to-work enrichment; standardized loadings 0.506–0.557 in the initial 51-item CFA) and CB2, CB7, and CB10 (family-domain thriving; loadings 0.560–0.622). The exclusion of these items resulted in improved model fit (*χ*^2^/df from 3.086 to 2.800; CFI from 0.837 to 0.880). A total of 44 items were retained for subsequent analyses. Item-level loadings and retained/deleted status are reported in Supplementary Table B1. The measurement-model analyses (CFA, competing models, and the common-method-bias comparison) were estimated in R (lavaan) with four first-order factors (FSSB, WFE, THR, ESB). The results indicated that the four-factor measurement model demonstrated acceptable to marginally acceptable fit (*χ*^2^ = 2508.566, df = 896, *χ*^2^/df = 2.800, RMSEA = 0.060, TLI = 0.874, CFI = 0.880, IFI = 0.881). In addition, the full structural model, with THR specified as a second-order construct comprising work thriving and family thriving, was estimated using the lavaan package in R. Model fit remained acceptable (*χ*^2^ = 2262.385, df = 894, *χ*^2^/df = 2.531, RMSEA = 0.055, CFI = 0.898, TLI = 0.892, SRMR = 0.046). As a sensitivity analysis linking the two specifications, a higher-order CFA for THR (44 items; two domain factors loading on a second-order THR factor, with FSSB, WFE, and ESB specified as first-order factors) showed acceptable fit (*χ*^2^ = 2262.385, df = 894, *χ*^2^/df = 2.531, RMSEA = 0.055, CFI = 0.898, TLI = 0.892, SRMR = 0.046), with substantial second-order loadings (0.939 and 0.863); the complete results are reported in Supplementary Table B2. Because the structural part of the mediation model is saturated (six structural paths among four latent variables), this higher-order CFA and the full SEM are equivalent reparameterizations of the same covariance structure, which is why their fit indices are identical. Because commonly used cutoff values for CFI/TLI should be interpreted as guidelines rather than rigid rules (Hu and Bentler, 1999; Marsh et al., 2004) and can be affected by model complexity and other conditions, model fit was evaluated holistically across indices and in comparison with alternative models (Shi et al., 2019). The four-factor model was significantly better than the three-factor, two-factor, and single-factor competing models (Table 1), supporting discriminant validity among the study variables.

Regarding convergent validity, standardized factor loadings ranged from 0.531 to 0.870, exceeding the recommended threshold of 0.50. Composite reliability (CR) values for all constructs exceeded 0.70, indicating good internal consistency. Regarding average variance extracted (AVE), all constructs met the recommended threshold except THR, whose AVE was 0.444, below the conventional 0.50 threshold (Table 2). Convergent validity evidence for THR is therefore mixed: the AVE value itself does not meet the common threshold, although the high composite reliability, the substantial second-order loadings, and the HTMT results (Table 3) provide supporting evidence. The square roots of AVE and Pearson correlation coefficients among the four variables are presented in Table 2 (Fornell and Larcker, 1981). The correlation coefficient between THR and WFE (0.679) was slightly higher than the square root of AVE for THR (0.666); the latent correlation between the WFE and THR factors was somewhat higher still (0.714; Supplementary Table B2), providing the more conservative comparison for discriminant validity. To further assess whether WFE and THR are empirically distinguishable, the study compared the baseline four-factor model with a constrained model fixing the WFE-THR correlation to 1.0 (Anderson and Gerbing, 1988). The constrained model fit significantly worse (Δ*χ*^2^ = 212.617, *p* < 0.001), supporting discriminant validity between WFE and THR. Moreover, the four-factor model demonstrated substantially better overall fit than competing models, suggesting acceptable discriminant validity at the overall construct level. Because THR was modeled as a second-order construct in the present study, its convergent validity was further supported by the substantial standardized loadings of the work thriving (0.939) and family thriving (0.863) dimensions on the higher-order THR factor, in addition to its composite reliability (CR = 0.931).

**Table 2:** Means, standard deviations, and correlations among study variables.

Variable	M	SD	1	2	3	4	5	6	7	8	9	10	11
1. Gender	1.60	0.49	—										
2. Age	1.28	0.45	−0.153^**^	—									
3. Education Level	3.08	0.75	0.068	−0.047	—								
4. Marital Status	1.47	0.50	−0.206^**^	0.372^**^	−0.065	—							
5. Position Level	1.54	0.50	−0.169^**^	0.482^**^	−0.042	0.445^**^	—						
6. Supervisor Gender	1.40	0.49	0.266^**^	−0.055	0.025	−0.049	−0.028	—					
7. Supervisor Age	2.94	0.82	0.076	0.178^**^	0.107^*^	0.187^**^	0.095^*^	−0.008	—				
8. FSSB	3.81	0.67	−0.167^**^	0.183^**^	−0.047	0.179^**^	0.302^**^	0.048	−0.017	(0.713)			
9. WFE	3.93	0.61	−0.099^*^	0.149^**^	−0.025	0.165^**^	0.274^**^	−0.121^**^	0.016	0.573^**^	(0.728)		
10. THR	3.82	0.59	−0.173^**^	0.201^**^	−0.034	0.243^**^	0.368^**^	−0.088^*^	−0.021	0.624^**^	0.679^**^	(0.666)	
11. ESB	3.47	0.99	−0.084	0.142^**^	−0.039	0.135^**^	0.246^**^	0.018	0.018	0.575^**^	0.529^**^	0.568^**^	(0.858)

*N* = 506; *M*, mean, SD, standard deviation. Values in parentheses on the diagonal represent the square roots of AVE; values below the diagonal represent Pearson correlation coefficients. ^*^p < 0.05, ^**^p < 0.01.

**Table 3:** HTMT ratios for discriminant validity.

Variable	FSSB	WFE	THR	ESB
FSSB	1			
WFE	0.622	1		
THR	0.670	0.742	1	
ESB	0.626	0.592	0.625	1

*N* = 506. HTMT, Heterotrait–Monotrait ratio of correlations (Henseler et al., 2015). All values were below the conservative threshold of 0.85; bootstrap 95% confidence intervals (5,000 resamples) excluded 1 for all pairs of constructs.

Nevertheless, the relatively high correlation between WFE and THR and AVE of 0.444 for THR indicate potential conceptual and empirical overlap. Serial mediation results involving both constructs should therefore be interpreted with caution and in light of the conceptual distinction discussed in the Theory section.

To further evaluate discriminant validity, we computed the HTMT (Henseler et al., 2015) based on the 44 retained items. As shown in Table 3, all HTMT values were below 0.85 and their bootstrap 95% confidence intervals excluded 1, supporting discriminant validity among the four constructs despite the relatively high WFE–THR correlation.

#### 4.1.2 Common method bias

Although multi-wave and multi-source data collection procedures reduce the risk of common method bias to some extent at the procedural level, it remains necessary to further examine potential common method bias statistically. As a preliminary diagnostic, Harman’s single-factor test was conducted using the final retained measurement items. Exploratory factor analysis of the 44 items revealed that the first unrotated factor accounted for 39.266% of the variance, which is below the conventional 40% threshold but close to it. Given the known limitations of this diagnostic (Podsakoff et al., 2003), the study further assessed common method bias using a CFA-based unmeasured latent method factor (ULMF) approach (Williams et al., 2010). Specifically, we compared the baseline measurement model (*χ*^2^ = 2508.566, df = 896) with an alternative model including a latent common method bias factor loading on all employee self-reported items (FSSB, WFE, and THR; method-factor variance fixed to 1). Although the *χ*^2^ difference was significant (*χ*^2^ = 2425.331, df = 856; Δ*χ*^2^(40) = 83.235, *p* < 0.001), the improvement in incremental fit indices was negligible (CFI = 0.880 vs. 0.883; TLI = 0.874 vs. 0.871; RMSEA = 0.060 in both models), and the method factor is a substantively uninterpreted latent variable; this pattern is not therefore considered evidence of a meaningful method effect. These results do not overturn the main substantive conclusions, but common method bias remains a concern because FSSB, WFE, and THR are employee self-reports. Interpretation of the mediation results therefore rests jointly on theory, design features, and bootstrap indirect effects rather than on a single common-method-bias diagnostic.

#### 4.1.3 Descriptive statistics and correlation analysis

Pearson correlation results among the main variables are shown in Table 2. FSSB was significantly positively correlated with ESB (*r* = 0.575, *p* < 0.01), WFE (*r* = 0.573, *p* < 0.01), and THR (*r* = 0.624, *p* < 0.01). WFE was significantly positively correlated with ESB (*r* = 0.529, *p* < 0.01) and THR (*r* = 0.679, *p* < 0.01). THR was also significantly positively correlated with ESB (*r* = 0.568, *p* < 0.01). Overall, the directions of the correlations were consistent with the hypotheses and provided preliminary support for subsequent regression and mediation analyses.

### 4.2 Hypothesis testing

To test the hypotheses while accounting for measurement error, a full latent structural equation model (SEM) was estimated using the lavaan package in R with the maximum likelihood estimator. The measurement model was specified as described above (44 retained items; THR as a second-order construct), and the structural model included the hypothesized paths among FSSB, WFE, THR, and ESB. Indirect effects were evaluated using bootstrap percentile 95% confidence intervals (5,000 resamples). The original hierarchical regression analyses are reported in the Supplementary material for transparency and reproducibility.

#### 4.2.1 Main effect of family-supportive supervisor behavior on employee stewardship behavior

To test the main effect, a latent model in which FSSB predicted ESB was estimated. FSSB was positively associated with ESB in this latent main-effect model (*β* = 0.649, *p* < 0.001; unstandardized estimate = 0.996, SE = 0.079), supporting Hypothesis 1. In the full serial mediation model, the total effect of FSSB on ESB was likewise significant (0.967, 95% CI = [0.830, 1.125]). Standardized path estimates of the full model are presented in Table 4 and illustrated in Figure 2; the original hierarchical regression results with demographic controls are reported in Supplementary Table C1.

**Table 4:** Standardized path estimates of the full latent structural model.

Path	*β*	SE	*z*	*p*
FSSB → WFE	0.602	0.044	13.84	***
FSSB → THR	0.381	0.053	7.15	***
WFE → THR	0.517	0.055	9.45	***
THR → ESB	0.328	0.078	4.22	***
WFE → ESB	0.112	0.066	1.71	0.087
FSSB → ESB	0.337	0.059	5.75	***

*N* = 506. Estimates are standardized; SE, standard error. Estimated with the full latent structural model in *R* (lavaan, ML); *z* and *p* are based on delta-method standard errors. Model fit: *χ*^2^ = 2262.385, df = 894, *χ*^2^/df = 2.531, RMSEA = 0.055, CFI = 0.898, TLI = 0.892, SRMR = 0.046. *** p<0.001, **p < 0.01, *p < 0.05.

**Figure 2:**
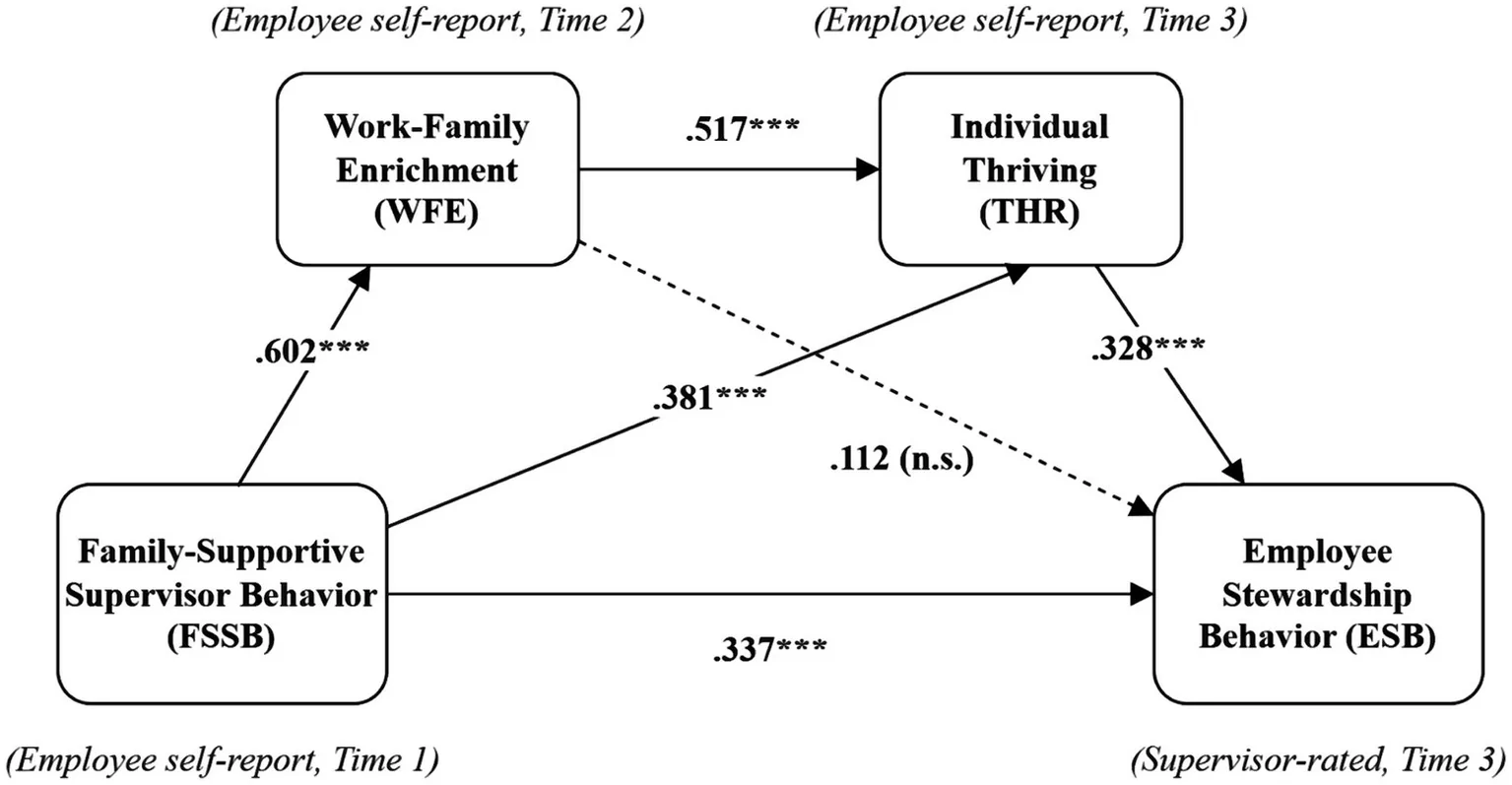
Standardized path estimates of the full latent structural model (*N* = 506). Solid lines indicate significant paths (***p < 0.001); the dashed line indicates the non-significant path from WFE to ESB (*β* = 0.112, *p* = 0.087).

#### 4.2.2 Mediating role of work-family enrichment

To examine the mediating role of WFE between FSSB and ESB, a latent mediation model was estimated in which FSSB predicted ESB through WFE. In this model, FSSB was positively associated with WFE (*β* = 0.626, *p* < 0.001), supporting Hypothesis 2a, and WFE was positively associated with ESB (*β* = 0.255, *p* < 0.001), supporting Hypothesis 2b. The indirect effect (FSSB → WFE → ESB) was 0.245 (95% CI = [0.138, 0.363]), excluding zero, based on bootstrap percentile confidence intervals with 5,000 resamples (Cheung, 2007). The direct effect remained significant (0.746, 95% CI = [0.597, 0.912]), indicating partial mediation. The indirect effect accounted for 24.70% of the total effect (Table 5). In this single-mediator model, Hypotheses 2a, 2b, and 2c were supported; however, as the focal full six-path model is the primary basis for hypothesis evaluation, these single-mediator results are reported as supplementary information. In the full six-path model the FSSB→WFE → ESB path was no longer significant once THR was simultaneously modeled (Table 4), indicating that WFE’s mediating role operates mainly through the serial pathway via THR rather than through an independent mediation mechanism (see the Serial Mediation section).

**Table 5:** Test results for the mediating effect of work-family enrichment (SEM).

Effect	Estimate	SE	LLCI	ULCI	Proportion of total effect
Total effect	0.991	0.078	0.850	1.156	100.00%
Direct effect	0.746	0.079	0.597	0.912	75.30%
Indirect effect	0.245	0.057	0.138	0.363	24.70%

Effects are unstandardized estimates from lavaan; 95% bootstrap percentile confidence intervals, 5,000 resamples.

In the full six-path structural model, in which WFE and THR simultaneously predicted ESB, the path from WFE to ESB was no longer significant (*β* = 0.112, *p* = 0.087). This occurred because WFE and THR are highly correlated (*r* = 0.679) and THR absorbed the shared variance in predicting ESB. This pattern indicates that the unique indirect pathway through WFE was not supported in the full model and that WFE contributed to ESB primarily through the serial pathway via THR, which is consistent with the serial mediation hypothesis.

#### 4.2.3 Mediating role of individual thriving

To examine the mediating role of THR, a latent mediation model was estimated in which FSSB predicted ESB through THR. In this model, FSSB was positively associated with THR (*β* = 0.715, *p* < 0.001), supporting Hypothesis 3a, and THR was positively associated with ESB (*β* = 0.385, *p* < 0.001), supporting Hypothesis 3b. The indirect effect (FSSB → THR → ESB) was 0.423 (95% CI = [0.274, 0.625]), with the confidence interval excluding zero, and the direct effect remained significant, indicating partial mediation. The indirect effect accounted for 42.79% of the total effect (Table 6). Therefore, Hypotheses 3a, 3b, and 3c were supported.

**Table 6:** Test results for the mediating effect of individual thriving (SEM).

Effect	Estimate	SE	LLCI	ULCI	Proportion of total effect
Total effect	0.989	0.078	0.847	1.153	100.00%
Direct effect	0.566	0.093	0.373	0.740	57.21%
Indirect effect	0.423	0.090	0.274	0.625	42.79%

Effects are unstandardized estimates from lavaan; 95% bootstrap percentile confidence intervals, 5,000 resamples.

#### 4.2.4 Serial mediation of work-family enrichment and individual thriving

The serial mediation test results are presented in Table 7 (full hierarchical regression results are reported in Supplementary Table C4). In the full six-path structural model, WFE was positively associated with THR (*β* = 0.517, *p* < 0.001), supporting Hypothesis 4a. The total indirect effect was 0.451 (95% CI = [0.306, 0.644]), accounting for 46.67% of the total effect (total effect = 0.967, 95% CI = [0.830, 1.125]). Path-specific tests showed that the serial pathway FSSB → WFE → THR → ESB was significant (0.156, 95% CI = [0.082, 0.255]), supporting Hypothesis 4b. The single-mediator pathway FSSB → THR → ESB was also significant (0.191, 95% CI = [0.091, 0.335]), whereas the single-mediator pathway FSSB → WFE → ESB was not significant in the full model (0.104, 95% CI = [−0.017, 0.229]), consistent with the substantial overlap between WFE and THR discussed above and with the separate single-mediator analyses reported in the preceding sections. As a robustness check, we also estimated an alternative ordering model (FSSB → THR → WFE → ESB) with the same measurement model (Table 8; the complete parameter estimates for both orderings are reported in Supplementary Table D1. Because the structural part of both models is saturated, the two orderings are equivalent reparameterizations of the same latent covariance structure, and their overall fit is identical by construction (both: *χ*^2^ = 2262.385, df = 894, *χ*^2^/df = 2.531, RMSEA = 0.055, CFI = 0.898, TLI = 0.892, SRMR = 0.046); overall fit therefore cannot distinguish the orderings. At the path level, the serial indirect effect was significant for FSSB → WFE → THR → ESB (0.156, SE = 0.044, 95% CI [0.082, 0.255]) but not for the reversed ordering FSSB → THR → WFE → ESB (0.075, SE = 0.046, 95% CI [−0.012, 0.167]). This pattern is consistent with the hypothesized ordering but does not independently establish it: the two orderings are observationally equivalent, so the difference between the two serial indirect effects reflects the reparameterization rather than differential empirical support, and the difference was not subjected to a formal statistical test; the comparison is therefore descriptive. The hypothesized ordering is preferred on theoretical grounds (the process–state–behavior logic and prior enrichment-to-thriving evidence) and is consistent with the measurement schedule (WFE at Time 2; THR at Time 3), but the design does not establish this ordering as a unique causal sequence.

**Table 7:** Test results for serial mediation effects.

Effect	Path	Estimate	SE	LLCI	ULCI	Proportion of total effect	Proportion of indirect effect
Total effect		0.967	0.075	0.830	1.125	100.00%	—
Direct effect		0.516	0.085	0.337	0.677	53.33%	—
Indirect effect	Path 1	0.104	0.062	−0.017	0.229	10.72%	22.98%
	Path 2	0.191	0.062	0.091	0.335	19.79%	42.39%
	Path 3	0.156	0.044	0.082	0.255	16.16%	34.63%
Total indirect effect		0.451	0.086	0.306	0.644	46.67%	100.00%

Effects are unstandardized estimates from lavaan; 95% bootstrap percentile confidence intervals, 5,000 resamples. Path 1 = FSSB → WFE → ESB; Path 2 = FSSB → THR → ESB; Path 3 = FSSB → WFE → THR → ESB. Minor differences between the total indirect effect and the sum of path-specific indirect effects are due to rounding.

**Table 8:** Comparison of the hypothesized and reversed ordering models.

Fit index/statistic	Hypothesized: FSSB→WFE → THR → ESB	Reversed: FSSB→THR → WFE → ESB
*χ*^2^ (df)	2262.385 (894)	2262.385 (894)
*χ*^2^/df	2.531	2.531
RMSEA	0.055	0.055
CFI	0.898	0.898
TLI	0.892	0.892
SRMR	0.046	0.046
Serial indirect effect	0.156	0.075
SE	0.044	0.046
95% CI	[0.082, 0.255]	[−0.012, 0.167]

*N* = 506. Both models were estimated with the same measurement model (44 items; THR specified as a second-order construct) using ML estimation; confidence intervals for the indirect effects are bootstrap percentile intervals based on 5,000 resamples. The structural part of both models is saturated, so the two orderings are equivalent reparameterizations of the same latent covariance structure and their overall fit is identical by construction. The serial indirect-effect entries are the same latent-SEM estimates reported in Table 7.

### 4.3 Multi-group analyses

To provide supplementary evidence regarding the robustness of the proposed theoretical model across different employee groups and to explore potential subgroup heterogeneity, multi-group analyses were conducted: structural-path comparisons and measurement-invariance tests were both estimated in R (lavaan) using the same 44-item measurement model as the focal structural model (Supplementary Table A11). Guided by COR and considering the actual distribution characteristics of the sample, eight variables were selected as grouping criteria, including gender, age, marital status, having dependent children, elder-care responsibility, position level, organizational tenure, and tenure with the current supervisor. These grouping variables reflect differences in individual resource endowment, family responsibility context, and occupational background. The multi-group analyses were conducted as supplementary tests to examine where, if at all, the proposed mechanism varies across employee subgroups. In addition, potential between-group differences in specific structural paths were explored to identify where heterogeneity in the resource-conversion process may occur.

Structural-path comparisons across groups presuppose that the measurement model operates equivalently across groups; we therefore first tested measurement invariance and then proceeded to structural-path equality tests, and we distinguish the two steps below. In the first step, for each grouping variable, a configural model (factor loadings freely estimated across groups) was compared with a metric model (factor loadings constrained equal across groups). ΔCFI was used as the primary criterion (values below 0.01), given the sensitivity of the *χ*^2^ difference test to sample size (Cheung and Rensvold, 2002). The *χ*^2^ difference test was significant at *p* < 0.05 for six of the eight groupings (age, marital status, elder-care responsibility, position level, organizational tenure, and tenure with the current supervisor), whereas ΔCFI values ranged from 0.000 to 0.002 across all eight groupings; both statistics are reported for each grouping in the Supplementary Table A11, and metric invariance was retained on the basis of the ΔCFI criterion. Scalar invariance was not tested, because our interest concerned structural paths rather than latent mean comparisons. It should be noted that the configural-model fit was modest in absolute terms (CFI = 0.842–0.870, below the conventional 0.90 benchmark), so the ΔCFI criterion was interpreted with caution and supplemented by the structural-model comparisons reported below.

In the second step, which is separate from the measurement-invariance tests, a configural structural model with freely estimated structural paths was compared with a structural-weights constrained model in which the focal structural paths were constrained to be equal across groups. Across all eight grouping variables, ΔCFI values were 0.000–0.001 and RMSEA values remained stable (0.063–0.065), indicating that constraining the structural paths did not meaningfully worsen model fit across subgroups; the *χ*^2^ difference test was significant only for age (Δ*χ*^2^(6) = 26.346, *p* < 0.001) and organizational tenure (Δ*χ*^2^(6) = 14.918, *p* = 0.021). Detailed model comparison results are reported in Supplementary Table A1.

Path-level differences were further examined using nested model *χ*^2^ difference tests, each imposing a single equality constraint (df = 1). A summary of significant between-group path differences is reported in Supplementary Table A2, and detailed unstandardized path coefficients for both groups (SEs in parentheses) and the Δ*χ*^2^ difference tests are reported in Supplementary Tables A3–A10. The results showed that significant subgroup differences were mainly concentrated in the upstream paths from FSSB to WFE and from FSSB to THR. Specifically, the FSSB→WFE path differed only across gender (Δ*χ*^2^(1) = 4.036, *p* = 0.045), whereas the FSSB→THR path differed across age (Δ*χ*^2^(1) = 13.195, *p* < 0.001), marital status (Δ*χ*^2^(1) = 8.627, *p* = 0.003), dependent child status (Δ*χ*^2^(1) = 5.851, *p* = 0.016), elder-care responsibility (Δ*χ*^2^(1) = 6.826, *p* = 0.009), position level (Δ*χ*^2^(1) = 4.861, *p* = 0.028), organizational tenure (Δ*χ*^2^(1) = 9.015, *p* = 0.003), and tenure with the current supervisor (Δ*χ*^2^(1) = 5.181, *p* = 0.023). In contrast, no significant differences were observed in the downstream paths from WFE and THR to ESB or in the path from WFE to THR. These path-level tests involved 48 between-group comparisons (8 grouping variables × 6 structural paths) without correction for multiple testing; the significant differences summarized in Supplementary Table A2 should therefore be interpreted as exploratory.

Overall, these supplementary analyses suggest that the downstream pathways from WFE and THR to ESB were relatively stable across employee subgroups, whereas the upstream pathways from FSSB showed subgroup heterogeneity. At the same time, the exploratory path-level comparisons tentatively suggest that subgroup heterogeneity may mainly emerge at the stage where family-supportive supervisory resources are perceived and converted into cross-domain and psychological resource gains. These findings suggest that employee characteristics may influence how family-supportive supervisory resources are perceived and converted into cross-domain and psychological resource gains. However, once WFE and THR are established, their relationships with ESB remain relatively consistent across subgroups. Thus, the multi-group results are consistent with relatively stable downstream pathways while indicating potential contextual variation in the upstream effectiveness of FSSB.

## 5 Discussion

### 5.1 Main findings and conclusions

In organizational contexts characterized by rapid digitalization, increasingly blurred work-family boundaries, and rising resource pressures, whether employees are willing to internalize organizational long-term interests as personal responsibilities despite potential risks and short-term costs represents a critical challenge for long-term organizational functioning. Grounded in COR, this study used multi-wave, multi-source employee-supervisor matched data to systematically examine the mechanisms through which FSSB influences ESB. Supplementary multi-group analyses were also conducted to examine the stability of the proposed resource-conversion and psychological-internalization mechanism across different employee subgroups, with particular attention to differences between upstream and downstream pathways.

(1) FSSB is positively associated with ESB, indicating that family-supportive supervision can function as an important supervisory resource that encourages employees to undertake long-term responsibility for organizational interests. This finding is consistent with evidence that FSSB can promote ESB through perceived insider status and proactive motivation (Wang and Zhao, 2020). However, the present study interprets this relationship from a resource-based perspective rather than primarily from a relational membership perspective. Prior research has also shown that FSSB is associated with positive employee outcomes (Guo et al., 2024; Pan, 2018), as well as performance (Chen et al., 2020), work attitudes (Jiang et al., 2015), well-being (Meng et al., 2024), and work-family experiences (Hammer et al., 2006). The present findings further suggest that the value of FSSB can extend to ESB, a long-term pro-organizational behavior centered on stewardship responsibility. From the perspective of COR, family-supportive supervisory resources help employees reduce resource strain, strengthen expectations of resource recoverability, and shift from short-term resource conservation toward long-term resource investment.

(2) WFE and THR explain how FSSB is transformed into ESB. The results show that FSSB is positively related to WFE, which is consistent with studies indicating that family-supportive supervision facilitates work-family enrichment by providing emotional, instrumental, and work-family management resources (Shen et al., 2022; Wang et al., 2022). The findings also show that FSSB is positively related to THR, which aligns with research suggesting that supportive organizational resources can foster employee thriving (Qin et al., 2022; Russo et al., 2018). In the full model, THR independently mediated the FSSB–ESB relationship, whereas the single-mediator path via WFE was non-significant once THR was accounted for; WFE’s contribution to ESB operated mainly through the serial pathway via THR, consistent with the serial mediation results discussed below. The high empirical overlap between WFE and THR (*r* = 0.679) implies that, once THR is entered into the full model, little unique variance remains for WFE to predict ESB, which accounts for the non-significance of the WFE → ESB path in the six-path model. Compared with studies that have mainly linked WFE to relatively proximal outcomes such as work engagement, work-family balance, job satisfaction, or performance (Deng et al., 2024; Jiang et al., 2015; Zhao et al., 2024), the present findings show that WFE also provides a resource basis for ESB through its pathway via THR. Similarly, while THR has often been discussed in relation to innovation, performance, and turnover intention (Abid et al., 2015; Gerbasi et al., 2015), the present study shows that thriving also matters for stewardship behavior oriented toward long-term organizational interests.

(3) WFE and THR form a serial mediation pathway between FSSB and ESB. This finding suggests that the association between FSSB and ESB is consistent with a resource-development sequence: family-supportive supervisory resources are first converted into cross-domain resource gains through WFE, then internalized into a stable psychological resource state through THR, and finally expressed as long-term stewardship behavior. This result is consistent with the resource enrichment perspective, which emphasizes the flow of positive resources between work and family domains (Han et al., 2020). It also resonates with studies showing that FSSB can promote thriving through WFE or related resource-development processes (Qin et al., 2022; Wang et al., 2022). However, unlike prior studies that mainly focused on well-being, individual prosperity, or work-related attitudes, the present study takes ESB as the final outcome and shows that the resource gains and psychological growth generated by FSSB can be further translated into behavior that protects the organization’s future-oriented interests.

A further point concerns marital status. Supplementary multi-group comparisons did not indicate that the core resource-conversion mechanism holds only for employees who have established a family. From a COR perspective, unmarried employees in contemporary labor markets may still allocate substantial resources to origin-family obligations, intimate partnerships, dual-career coordination, and anticipated eldercare. Marital status labels therefore do not correspond one-to-one to family-role resource load. Consistent with the multi-group pattern of greater upstream heterogeneity and relatively stable downstream paths, marital-status differences were confined to the upstream FSSB → THR path (Δ*χ*^2^(1) = 8.627, *p* = 0.003; Supplementary Table A2), and the absence of marital-status differences on the downstream stewardship paths is interpretable without assuming that WFE is meaningful only for married parents. External validity to populations with more intensive childcare or eldercare demands nonetheless remains bounded by the still relatively young sample profile.

Overall, from a work-family interaction perspective, this study shows that FSSB provides perceptible and transformable supervisory resources that help employees move from short-term resource conservation toward long-term responsibility internalization. WFE and THR jointly constitute the resource-conversion and psychological-internalization mechanisms through which FSSB promotes ESB. In organizational contexts characterized by increasingly blurred work-family boundaries and rising resource pressures, implementing family-supportive supervisory practices can therefore represent an important pathway for fostering employee stewardship behavior and supporting long-term organizational functioning.

An important alternative explanation is shared relationship quality or liking. Employees who have higher-quality exchanges with supervisors may rate FSSB more favorably, and the same supervisors may rate those employees higher on ESB, potentially inflating the FSSB–ESB association. Temporal separation and multi-source measurement partially mitigate but do not eliminate leader–member exchange or relationship-quality confounds. We therefore do not claim that the proposed resource mechanism is unique or that all alternative relational explanations are ruled out.

### 5.2 Theoretical implications

The study makes three primary theoretical contributions.

First, building on evidence that FSSB can be related to ESB through perceived insider status and proactive motivation (Wang and Zhao, 2020), we introduce a COR-based resource-transformation perspective. We do not claim to be the first to document an FSSB–ESB link; the contribution is to shift from a primarily relational or motivational logic toward a resource-based logic in which ESB is understood as long-term resource investment under conditions of resource security.

Second, building on evidence that FSSB is associated with improved work-family experiences (Jiang et al., 2015), and employee thriving (Russo et al., 2018), this study specifies a sequential transmission process—supervisory resources → cross-domain enrichment (WFE) → psychological thriving (THR) → stewardship-oriented behavior (ESB). This serial mechanism shows that family-supportive supervision can extend beyond proximal work–family strain reduction or short-term attitudes to responsibility-oriented behavior that supports long-term organizational interests.

Third, by integrating WFE and THR within COR, we connect employee resource development across work and family domains with long-term organizational functioning. Supportive supervisory practices may matter not only by preventing depletion, but also by enabling resource-gain processes that help employees assume stewardship responsibilities beyond immediate task fulfillment.

### 5.3 Practical implications

Against the broader backdrop of intensified work-family pressures, organizations increasingly need to help employees balance work demands with family responsibilities. Such strain may undermine both individual development and long-term organizational functioning. Based on the findings, this study offers four targeted practical implications for organizational management. However, FSSB is an important lever for ESB but not a sufficient condition; fairness, trust, psychological safety, organizational culture, and incentive systems also matter.

First, organizations may treat FSSB as an important supervisory practice for cultivating employee stewardship. The results suggest that when organizations seek to develop employees who are willing to safeguard collective organizational interests and share risks under uncertainty, FSSB can serve as a substantive lever for strengthening ESB. Supervisors’ understanding of and support for employees’ family responsibilities may not only improve short-term work-family experiences, but also replenish critical resources and enhance employees’ willingness to treat organizational development as a long-term investment. Accordingly, organizations may incorporate FSSB into leadership development systems. Through institutional guidance and competency-based training, managers can be supported to consistently enact emotional support, instrumental support, role modeling and flexible work-family management practices, thereby fostering employees’ long-term responsibility commitment. To make this actionable, training and evaluation can be dimension-specific. For emotional support, training may focus on active listening about family demands and destigmatizing leave and recovery needs, with brief subordinate ratings of supervisor empathy and approachability in manager development reviews. For instrumental support, training may cover task reprioritization, temporary coverage, and flexible scheduling when family emergencies arise, with metrics such as response timeliness to flexibility requests, documented coverage plans, and team overtime spikes after leave. For role modeling, supervisors should demonstrate reasonable after-hours norms and visible use of family-friendly policies, with 360-degree or peer indicators of boundary-consistent leader behavior and policy-use signaling without career penalty. For creative work-family management, organizations may emphasize predictable scheduling, cross-training for coverage, and outcome-oriented (not presenteeism-based) performance, with workshop completion plus 30–60 day action-plan follow-up and team policy awareness or schedule predictability scores.

Second, for relatively young employees—who form a large share of many knowledge workforces—organizations should jointly support efficiency and balance: clear priorities, predictable workloads, recovery-supportive practices, and result-oriented evaluation rather than pure face-time. Such practices can strengthen perceived resource security that our model links to enrichment, thriving, and stewardship-oriented behavior.

Third, organizations should strengthen work-life balance practices that promote WFE. The findings show that WFE contributes to ESB mainly through THR, with which it forms a serial resource pathway linking family-supportive supervision to stewardship behavior. This suggests that work-life balance practices should not be viewed only as welfare-oriented arrangements for reducing strain, but also as resource-enrichment mechanisms that help employees transform resources across work and family domains. Practically, organizations can support WFE through flexible scheduling, predictable task allocation, reasonable workload planning, family-friendly leave policies, remote work options, and autonomy in managing work-family boundaries. These practices can help employees experience work and family roles as mutually reinforcing rather than purely competing domains, thereby enhancing resource security and increasing their capacity to engage in long-term stewardship behavior.

Fourth, organizations should cultivate employees’ individual thriving by supporting sustained vitality and learning. The findings show that THR is an important psychological mechanism linking FSSB and WFE to ESB. This suggests that work-family resource gains are more likely to translate into long-term stewardship behavior when employees possess sustained energy, learning opportunities, and developmental momentum. Therefore, organizations should provide recovery-supportive practices, developmental feedback, learning opportunities, autonomy-supportive work design, and a culture that values both family responsibility and employee growth. These practices can help employees maintain vitality and learning under work-family pressures, thereby strengthening the psychological foundation for stable ESB oriented toward long-term organizational interests.

### 5.4 Limitations and future research

Grounded in COR, this study used a multi-wave, multi-source design to examine the mechanisms linking FSSB to ESB, yielding both theoretical and practical implications. Nevertheless, several limitations warrant consideration and point to future research.

First, although the multi-wave and multi-source design provides temporal separation and supervisor-rated ESB reduces reliance on a single employee-reported source for the outcome, the study remains non-experimental and questionnaire-based. Therefore, the findings do not establish causal relationships. The two-week intervals were selected to balance temporal separation with feasibility and retention in a three-wave matched design, but they may be too short to capture enduring dynamics in relatively stable states such as WFE and THR. In addition, THR and ESB were both measured at Time 3, which further limits strong temporal claims regarding the final path in the serial mediation model. Future research may use longer lags, intensive longitudinal designs, experiments, additional raters, or behavioral indicators of ESB. Moreover, although the WFE → THR order is theoretically preferred because WFE represents a cross-domain resource-conversion process whereas THR represents a psychological resource state of vitality and learning, the present design does not establish this order as a unique causal sequence. Reverse or reciprocal relationships between WFE and THR remain conceivable. Although we estimated a reversed-order alternative model as a robustness check (see Serial Mediation section), stronger tests require longer-term longitudinal, intensive longitudinal, or experimental designs.

Second, although the age distribution reported in the submitted manuscript has been corrected, the sample remains relatively young. Specifically, 71.94% of participants were aged 35 years or below, whereas 28.06% were older than 35. The earlier statement that all participants were 35 years of age or younger was incorrect and has been removed. In addition, 52.96% of participants were unmarried. Therefore, generalizability to populations with more intensive childcare or eldercare responsibilities should be interpreted cautiously. Future research should replicate the model across broader age ranges and more diverse family-life stages. ESB was also measured using a three-item scale originally developed in a family-business context. Although the items capture individual-level prioritization of long-term organizational interests and have been used in broader organizational settings, three items cannot fully represent the multidimensional nature of stewardship behavior, and independent validity evidence in non-family Chinese organizations remains limited. Future research should use fuller ESB measures and, where possible, combine supervisor ratings with behavioral or multi-source indicators. The absence of wave-specific retention counts, per-reason exclusion counts, and sampling-frame information (the number, industry, and size of participating organizations) further limits the evaluation of attrition and the assessment of generalizability. In addition, the seven deleted items were identified from the CFA results of the same dataset rather than from an *a priori* measurement plan; all seven belong to family-side dimensions (family-to-work enrichment and family-domain thriving), which reduces family-side coverage, and the second-order THR factor is indicated by only two first-order domain factors, so the higher-order structure is just-identified at that level. These features should be considered when interpreting the family-side constructs.

Third, this study identified WFE and THR as resource-conversion and psychological-internalization mechanisms linking FSSB to ESB, but it did not formally examine when these mechanisms become stronger or weaker. The supplementary multi-group analyses provided only preliminary clues that subgroup differences may exist in the upstream paths from FSSB to WFE and THR. Future research could build on these findings by testing potential moderators, such as career stage, organizational tenure, tenure with the current supervisor, family responsibility status, or gender. Moderated mediation models may further clarify the boundary conditions under which FSSB is more strongly associated with cross-domain resource gains, psychological thriving, and long-term stewardship-oriented behavior. In addition, WFE and THR are conceptually distinct but empirically related. The relatively high correlation between WFE and THR and the AVE of 0.444 for THR indicate potential conceptual and empirical overlap. Although the multi-wave design, Harman’s single-factor test, and the ULMF analysis mitigate but do not eliminate concerns about common method bias, FSSB, WFE, and THR were all employee self-reports; common method bias therefore cannot be fully ruled out. Following best-practice recommendations on control variables (Bernerth and Aguinis, 2016), the study deliberately distinguished statistical controls from conceptually overlapping alternative mechanisms. Moreover, the study did not directly measure several theoretically relevant alternative explanations, such as leader–member exchange, general supervisor support, perceived organizational support, organizational identification, job demands, and relationship quality. Thus, the proposed resource-based mechanism should not be interpreted as the only possible explanation.

Fourth, the original data-collection protocol did not record whether supervisors were instructed to rate only one focal employee or were allowed to rate multiple participating employees. Because unique supervisor identifiers were not retained in the dataset, whether nesting occurred cannot be determined, and the degree of clustering (the number of supervisors and the number of employees rated by each) cannot be reconstructed; intraclass correlations and cluster-robust standard errors therefore cannot be computed. To the extent that a given supervisor rated multiple employees, the standard errors of the supervisor-rated outcome (ESB) and of the paths involving ESB may be underestimated and statistical significance may be inflated. This caveat applies to the inferential results of the present study and is not merely a direction for future research; future studies should nonetheless collect supervisor identifiers, report intraclass correlations, and use multilevel models or cluster-robust standard errors where appropriate.

## Data Availability

The de-identified data supporting the conclusions of this article are available from the corresponding author upon reasonable request. The archived analysis scripts and outputs that reproduce all statistics reported in the manuscript are provided as Supplementary material.
