# Supplementary material for: From family-supportive supervisor behavior to employee stewardship behavior: the serial mediating roles of work-family enrichment and individual thriving
Source: Front Psychol. 2026 Oct 23;17:1878413. doi: 10.3389/fpsyg.2026.1878413 (PMC13739481; doi:10.3389/fpsyg.2026.1878413)
Supplement: Supplementary file 1 [file Data_Sheet_1.docx]

Supplementary Material

# 1 Supplementary Material A

Supplementary Table A1 presents the model comparison results between configural models and structural-weights constrained models across eight grouping variables. The model comparison analyses were conducted in R (lavaan) using the same 44-item measurement model as the focal structural model and the measurement-invariance tests in Table A11, with THR specified as a second-order construct. For each grouping variable, the configural model allowed structural paths to be freely estimated across groups, whereas the constrained model fixed the six focal structural paths to be equal across groups. Because the structural part of the model is saturated, the configural rows are numerically identical to the configural models reported in Table A11. ΔCFI and Δχ² were used to evaluate whether imposing equality constraints substantially worsened model fit.

**Table A1** Model Fit Comparison Between Configural and Structural Constrained Models Across Groups

| **Grouping Variable** | **Model** | **χ²** | **df** | **χ²/df** | **CFI** | **TLI** | **RMSEA** | **ΔCFI** | **Δχ²(6)** | **p** |
| --- | --- | --- | --- | --- | --- | --- | --- | --- | --- | --- |
| Gender | Configural | 3652.339 | 1788 | 2.043 | .865 | .857 | .064 | -- | -- | -- |
|  | Constrained | 3662.318 | 1794 | 2.041 | .864 | .857 | .064 | .000 | 9.979 | .126 |
| Age | Configural | 3628.234 | 1788 | 2.029 | .865 | .857 | .064 | -- | -- | -- |
|  | Constrained | 3654.580 | 1794 | 2.037 | .863 | .856 | .064 | .001 | 26.346 | <.001 |
| Marital Status | Configural | 3642.674 | 1788 | 2.037 | .864 | .856 | .064 | -- | -- | -- |
|  | Constrained | 3652.985 | 1794 | 2.036 | .863 | .856 | .064 | .000 | 10.311 | .112 |
| Dependent Child Status | Configural | 3673.871 | 1788 | 2.055 | .863 | .855 | .065 | -- | -- | -- |
|  | Constrained | 3682.941 | 1794 | 2.053 | .862 | .855 | .065 | .000 | 9.070 | .170 |
| Elder-Care Responsibility | Configural | 3565.931 | 1788 | 1.994 | .870 | .863 | .063 | -- | -- | -- |
|  | Constrained | 3576.534 | 1794 | 1.994 | .870 | .863 | .063 | .000 | 10.603 | .101 |
| Position Level | Configural | 3668.481 | 1788 | 2.052 | .854 | .845 | .064 | -- | -- | -- |
|  | Constrained | 3675.418 | 1794 | 2.049 | .853 | .845 | .064 | .000 | 6.937 | .327 |
| Organizational Tenure | Configural | 3605.539 | 1788 | 2.017 | .859 | .851 | .063 | -- | -- | -- |
|  | Constrained | 3620.457 | 1794 | 2.018 | .858 | .850 | .063 | .001 | 14.918 | .021 |
| Tenure with Current Supervisor | Configural | 3658.295 | 1788 | 2.046 | .842 | .832 | .064 | -- | -- | -- |
|  | Constrained | 3669.163 | 1794 | 2.045 | .841 | .832 | .064 | .000 | 10.867 | .093 |

Note. N = 506. Both models were estimated in R (lavaan; ML estimator) with the same measurement model as the focal SEM (44 items; THR specified as a second-order construct). The constrained model fixes the six structural paths (FSSB → WFE, WFE → THR, FSSB → THR, THR → ESB, FSSB → ESB, WFE → ESB) to be equal across the two groups of each grouping variable. Configural rows are numerically identical to the configural models in Table A11 because the structural part of the model is saturated. ΔCFI = CFI(configural) − CFI(constrained), computed from unrounded values. Δχ² has df = 6 (six equality constraints).

Supplementary Table A2 summarizes statistically significant between-group path differences identified through nested model χ² difference tests (one equality constraint per test), estimated in the same lavaan framework as Table A1. Only paths with p < 0.05 are reported.

**Table A2** Summary of Significant Between-Group Path Differences

| **Grouping Variable** | **Path** | **Δχ²(1)** | **p** |
| --- | --- | --- | --- |
| Gender | FSSB → WFE | 4.036 | .045 |
| Age | FSSB → THR | 13.195 | <.001 |
| Marital Status | FSSB → THR | 8.627 | .003 |
| Dependent Child Status | FSSB → THR | 5.851 | .016 |
| Elder-Care Responsibility | FSSB → THR | 6.826 | .009 |
| Position Level | FSSB → THR | 4.861 | .028 |
| Organizational Tenure | FSSB → THR | 9.015 | .003 |
| Tenure with Current Supervisor | FSSB → THR | 5.181 | .023 |

Note. The table summarizes the between-group differences that reached p < 0.05 in the nested model χ² difference tests (df = 1), estimated in the same lavaan framework as Table A1. The complete unstandardized path coefficients for both groups of every grouping variable are reported in Tables A3–A10. These comparisons are part of 48 between-group tests (8 grouping variables × 6 structural paths) conducted without correction for multiple testing and should be interpreted as exploratory.

Supplementary Tables A3-A10 provide detailed statistical results for the multi-group analyses reported in Section 4.3. These analyses were conducted in R (lavaan) using the same 44-item measurement model as the focal structural model, with THR specified as a second-order construct. The tables present unstandardized structural-path coefficients (SEs in parentheses) for both groups, and a nested model χ² difference test for each path (df = 1); a significant Δχ² indicates a statistically significant between-group difference for the corresponding path (^***^p < 0.001, ^**^p < 0.01, ^*^p < 0.05).

**Table A3** Unstandardized Path Coefficients and Between-Group Difference Tests by Age

| **Path** | **Lower age group (N=364)** | **Higher age group (N=142)** | **Δχ²(1)** | **p** |
| --- | --- | --- | --- | --- |
| FSSB→WFE | 0.582 (0.063) | 0.748 (0.114) | 1.740 | .187 |
| WFE→THR | 0.386 (0.049) | 0.240 (0.063) | 2.352 | .125 |
| FSSB→THR | 0.239 (0.043) | 0.607 (0.109) | 13.195 | <.001 |
| THR→ESB | 0.684 (0.167) | 0.586 (0.260) | 0.170 | .680 |
| FSSB→ESB | 0.565 (0.107) | 0.337 (0.151) | 1.825 | .177 |
| WFE→ESB | 0.168 (0.098) | 0.150 (0.162) | 0.013 | .908 |

**Table A4** Unstandardized Path Coefficients and Between-Group Difference Tests by Gender

| **Path** | **Male (N=203)** | **Female (N=303)** | **Δχ²(1)** | **p** |
| --- | --- | --- | --- | --- |
| FSSB→WFE | 0.531 (0.064) | 0.749 (0.094) | 4.036 | .045 |
| WFE→THR | 0.344 (0.048) | 0.473 (0.076) | 2.220 | .136 |
| FSSB→THR | 0.231 (0.045) | 0.387 (0.073) | 3.547 | .060 |
| THR→ESB | 0.620 (0.178) | 0.828 (0.215) | 1.176 | .278 |
| FSSB→ESB | 0.501 (0.110) | 0.548 (0.120) | 0.117 | .733 |
| WFE→ESB | 0.115 (0.110) | 0.206 (0.125) | 0.513 | .474 |

**Table A5**. Unstandardized Path Coefficients and Between-Group Difference Tests by Position Level

| **Path** | **Frontline (N=234)** | **Management (N=272)** | **Δχ²(1)** | **p** |
| --- | --- | --- | --- | --- |
| FSSB→WFE | 0.539 (0.078) | 0.623 (0.076) | 0.613 | .434 |
| WFE→THR | 0.358 (0.054) | 0.339 (0.062) | 0.052 | .820 |
| FSSB→THR | 0.194 (0.046) | 0.364 (0.064) | 4.861 | .028 |
| THR→ESB | 0.621 (0.195) | 0.728 (0.221) | 0.246 | .620 |
| FSSB→ESB | 0.535 (0.115) | 0.544 (0.124) | 0.004 | .952 |
| WFE→ESB | 0.152 (0.107) | 0.207 (0.138) | 0.157 | .692 |

**Table A6** Unstandardized Path Coefficients and Between-Group Difference Tests by Organizational Tenure

| **Path** | **Short-tenure employees (N=292)** | **Long-tenure employees (N=214)** | **Δχ²(1)** | **p** |
| --- | --- | --- | --- | --- |
| FSSB→WFE | 0.541 (0.071) | 0.678 (0.088) | 1.544 | .214 |
| WFE→THR | 0.361 (0.053) | 0.348 (0.065) | 0.023 | .881 |
| FSSB→THR | 0.206 (0.046) | 0.460 (0.077) | 9.015 | .003 |
| THR→ESB | 0.688 (0.182) | 0.637 (0.220) | 0.060 | .807 |
| FSSB→ESB | 0.557 (0.116) | 0.497 (0.132) | 0.152 | .696 |
| WFE→ESB | 0.145 (0.105) | 0.219 (0.137) | 0.283 | .595 |

**Table A7** Unstandardized Path Coefficients and Between-Group Difference Tests by Tenure with Current Supervisor

| **Path** | **Short tenure (N=272)** | **Long tenure (N=234)** | **Δχ²(1)** | **p** |
| --- | --- | --- | --- | --- |
| FSSB→WFE | 0.505 (0.077) | 0.650 (0.088) | 1.605 | .205 |
| WFE→THR | 0.353 (0.051) | 0.328 (0.073) | 0.073 | .787 |
| FSSB→THR | 0.186 (0.046) | 0.380 (0.077) | 5.181 | .023 |
| THR→ESB | 0.728 (0.198) | 0.486 (0.232) | 1.071 | .301 |
| FSSB→ESB | 0.579 (0.120) | 0.382 (0.129) | 1.604 | .205 |
| WFE→ESB | 0.183 (0.104) | 0.113 (0.154) | 0.214 | .644 |

**Table A8** Unstandardized Path Coefficients and Between-Group Difference Tests by Dependent Child Status

| **Path** | **No (N=281)** | **Yes (N=225)** | **Δχ²(1)** | **p** |
| --- | --- | --- | --- | --- |
| FSSB→WFE | 0.677 (0.086) | 0.577 (0.070) | 0.834 | .361 |
| WFE→THR | 0.412 (0.080) | 0.397 (0.050) | 0.025 | .874 |
| FSSB→THR | 0.421 (0.079) | 0.214 (0.044) | 5.851 | .016 |
| THR→ESB | 0.732 (0.210) | 0.515 (0.167) | 1.225 | .268 |
| FSSB→ESB | 0.603 (0.128) | 0.515 (0.108) | 0.365 | .546 |
| WFE→ESB | 0.294 (0.148) | 0.147 (0.105) | 1.062 | .303 |

**Table A9** Unstandardized Path Coefficients and Between-Group Difference Tests by Elder-Care Responsibility

| **Path** | **No (N=232)** | **Yes (N=274)** | **Δχ²(1)** | **p** |
| --- | --- | --- | --- | --- |
| FSSB→WFE | 0.579 (0.078) | 0.623 (0.071) | 0.179 | .672 |
| WFE→THR | 0.414 (0.060) | 0.326 (0.055) | 1.154 | .283 |
| FSSB→THR | 0.202 (0.049) | 0.405 (0.063) | 6.826 | .009 |
| THR→ESB | 0.652 (0.174) | 0.588 (0.202) | 0.114 | .736 |
| FSSB→ESB | 0.555 (0.115) | 0.476 (0.115) | 0.331 | .565 |
| WFE→ESB | 0.196 (0.106) | 0.150 (0.126) | 0.125 | .724 |

**Table A10** Unstandardized Path Coefficients and Between-Group Difference Tests by Marital Status

| **Path** | **Unmarried (N=268)** | **Married (N=238)** | **Δχ²(1)** | **p** |
| --- | --- | --- | --- | --- |
| FSSB→WFE | 0.585 (0.075) | 0.609 (0.074) | 0.053 | .818 |
| WFE→THR | 0.368 (0.051) | 0.453 (0.077) | 0.854 | .355 |
| FSSB→THR | 0.194 (0.044) | 0.436 (0.074) | 8.627 | .003 |
| THR→ESB | 0.534 (0.168) | 0.752 (0.215) | 1.236 | .266 |
| FSSB→ESB | 0.507 (0.108) | 0.617 (0.123) | 0.618 | .432 |
| WFE→ESB | 0.166 (0.107) | 0.203 (0.139) | 0.071 | .790 |

Supplementary Table A11 presents the measurement invariance tests (configural vs. metric models) for each of the eight grouping variables, estimated in R (lavaan) with the same measurement model as the focal structural model. For each grouping, a configural model with factor loadings freely estimated across groups was compared with a metric model in which factor loadings were constrained to be equal across groups. The table reports the complete fit statistics of the configural and metric models for each grouping variable, followed by the difference tests. ΔCFI was used as the primary criterion given the sensitivity of the chi-square difference test to sample size (Cheung and Rensvold, 2002). Across all eight groupings, ΔCFI values ranged from 0.000 to 0.002, supporting metric invariance.

**Table A11** Measurement Invariance Tests (Configural vs. Metric)

| **Group** | **n1/n2** | **Configural χ² (df=1788)** | **RMSEA** | **CFI** | **TLI** | **Metric χ² (df=1828)** | **RMSEA** | **CFI** | **TLI** | **Δχ²(40)** | **p** | **ΔCFI** |
| --- | --- | --- | --- | --- | --- | --- | --- | --- | --- | --- | --- | --- |
| Gender | 203/303 | 3652.339 | 0.064 | 0.865 | 0.857 | 3694.794 | 0.064 | 0.864 | 0.860 | 42.455 | 0.366 | 0.000 |
| Age | 364/142 | 3628.234 | 0.064 | 0.865 | 0.857 | 3687.245 | 0.063 | 0.863 | 0.858 | 59.011 | 0.027 | 0.001 |
| Marital | 268/238 | 3642.674 | 0.064 | 0.864 | 0.856 | 3712.234 | 0.064 | 0.861 | 0.857 | 69.560 | 0.003 | 0.002 |
| Child | 281/225 | 3673.871 | 0.065 | 0.863 | 0.855 | 3728.664 | 0.064 | 0.862 | 0.857 | 54.794 | 0.060 | 0.001 |
| Elder | 232/274 | 3565.931 | 0.063 | 0.870 | 0.863 | 3622.824 | 0.062 | 0.869 | 0.864 | 56.892 | 0.040 | 0.001 |
| Position | 234/272 | 3668.481 | 0.064 | 0.854 | 0.845 | 3731.603 | 0.064 | 0.852 | 0.847 | 63.122 | 0.011 | 0.002 |
| TenureOrg | 292/214 | 3605.539 | 0.063 | 0.859 | 0.851 | 3664.318 | 0.063 | 0.857 | 0.852 | 58.779 | 0.028 | 0.001 |
| TenureSup | 272/234 | 3658.295 | 0.064 | 0.842 | 0.832 | 3726.466 | 0.064 | 0.839 | 0.833 | 68.171 | 0.004 | 0.002 |

Note. Configural model: factor loadings freely estimated across groups. Metric model: factor loadings constrained equal across groups. Models estimated in lavaan (ML) with the same measurement model as the SEM (44 items; THR second-order). ΔCFI = CFI(configural) − CFI(metric); values of ΔCFI below 0.01 support metric invariance (Cheung and Rensvold, 2002) and were used as the primary criterion given the sensitivity of the chi-square difference test to sample size. Scalar invariance was not tested (no latent mean comparisons were of interest).

# 2 Supplementary Material B

Supplementary Table B1 provides item-level information for all items of the four measurement scales, including retained/deleted status, standardized factor loadings, average variance extracted (AVE), and composite reliability (CR).

Table B1. Item-Level Information, Retained/Deleted Status, Loadings, AVE, and CR

| **Item** | **Construct** | **Dimension** | **Status** | **Std. loading** | **AVE** | **CR** |
| --- | --- | --- | --- | --- | --- | --- |
| BB4 | WFE | Family-to-work enrichment | Deleted | 0.557 | — | — |
| BB5 | WFE | Family-to-work enrichment | Deleted | 0.506 | — | — |
| BB6 | WFE | Family-to-work enrichment | Deleted | 0.546 | — | — |
| BB7 | WFE | Family-to-work enrichment | Deleted | 0.529 | — | — |
| CB2 | THR | Family learning | Deleted | 0.622 | — | — |
| CB7 | THR | Family vitality | Deleted | 0.560 | — | — |
| CB10 | THR | Family vitality | Deleted | 0.570 | — | — |
| AA1–AD4 | FSSB | Emotional/Instrumental/Role modeling/Creative W-FM | Retained (14) | 0.656–0.747 | 0.509 | 0.935 |
| BA1–BA7, BB1–BB3 | WFE | Work-to-family/Family-to-work enrichment | Retained (10) | 0.531–0.847 | 0.530 | 0.916 |
| CA1–CA10, CB1/CB3/CB4/CB5/CB6/CB8/CB9 | THR | Work/family vitality + learning | Retained (17) | 0.576–0.763 | 0.444 | 0.931 |
| DA1–DA3 | ESB | Stewardship behavior | Retained (3) | 0.851–0.870 | 0.736 | 0.893 |

Note. Std. loading = standardized factor loading. Standardized loadings are from the initial 51-item four-factor CFA for the deleted items and from the final 44-item CFA for the retained items. AVE and CR are computed on the 44 retained items, consistent with the manuscript. Items BB4-BB7 and CB2, CB7, CB10 were deleted because of their relatively low standardized loadings in the initial 51-item CFA, and their removal substantially improved model fit (χ²/df from 3.086 to 2.800; CFI from 0.837 to 0.880).

To connect the first-order measurement model in Table B1 with the second-order specification used in the structural model, Table B2 reports a sensitivity analysis in which THR was specified as a higher-order factor.

Second-order loadings (standardized): work thriving → THR = 0.939; family thriving → THR = 0.863. Standardized correlations among first-order factors: FSSB–WFE = 0.602; FSSB–THR = 0.665; FSSB–ESB = 0.631; WFE–THR = 0.714; WFE–ESB = 0.560; THR–ESB = 0.620.

Table B2. Higher-Order CFA for THR (Sensitivity Analysis)

| **Statistic** | **Value** |
| --- | --- |
| χ² (df) | 2262.385 (894) |
| χ²/df | 2.531 |
| RMSEA [90% CI] | 0.055 [0.052, 0.058] |
| CFI | 0.898 |
| TLI | 0.892 |
| SRMR | 0.046 |

Note. N = 506. 44 retained items; THR specified as a second-order factor indicated by work thriving (CA1–CA10) and family thriving (CB1, CB3–CB6, CB8, CB9); FSSB, WFE, and ESB specified as first-order factors. Estimated in lavaan (ML). Because the structural part of the full SEM is saturated, this higher-order CFA and the full SEM are equivalent reparameterizations of the same covariance structure, which is why their fit indices are identical. This analysis links the first-order CFA reported in Table 1 of the main text (used to validate the measurement model and establish construct distinctiveness) with the higher-order specification used in the SEM.

The following are the measurement items adopted.

**Family-Supportive Supervisor Behavior (FSSB) Scale**

| **Items** | **Questionnaire items** |
| --- | --- |
| Emotional support | 1. My supervisor is willing to listen to my problems in juggling work and nonwork life. 2. My supervisor takes the time to learn about my personal needs. 3. My supervisor makes me feel comfortable talking to him or her about my conflicts between work and nonwork. 4. My supervisor and I can talk effectively to solve conflicts between work and nonwork issues. |
| Instrumental support | 5. I can depend on my supervisor to help me with scheduling conflicts if I need it. 6. I can rely on my supervisor to make sure my work responsibilities are handled when I have unanticipated nonwork demands. 7. My supervisor works effectively with workers to creatively solve conflicts between work and nonwork. |
| Role model | 8. My supervisor is a good role model for work and nonwork balance. 9. My supervisor demonstrates effective behaviors in how to juggle work and nonwork balance. 10. My supervisor demonstrates how a person can jointly be successful on and off the job. |
| Creative work-family management | 11. My supervisor thinks about how the work in my department can be organized to jointly benefit employees and the company. 12. My supervisor asks for suggestions to make it easier for employees to balance work and nonwork demands. 13. My supervisor is creative in reallocating job duties to help my department work better as a team. 14. My supervisor is able to manage the department as a whole team to enable everyone's needs to be met. |

**Work-Family Enrichment (WFE) Scale**

| **Items** | **Questionnaire items** |
| --- | --- |
| Work → Family Enrichment | 1. My work helps me learn to listen to and understand perspectives different from my own, which helps me interact better with my family. 2. My work broadens my horizons and helps me acquire new information and knowledge, making my family see me as a more interesting person. 3. My work helps me develop interpersonal and communication skills, enabling me to perform my family role more effectively. 4. The positive mood I experience at work carries over after work, making me more proactive in interacting with my family. 5. My work achievements make me feel happy, improve my interactions with my family, and allow me to better appreciate the happiness of family life. 6. My work makes me feel that my life is fulfilling, allowing me to better enjoy the happiness brought by being with my family. 7. My work gives me a sense of accomplishment and satisfaction, making my family see me as a positive and optimistic person. |
| Family → Work Enrichment | 8. My interactions with my family help me better understand how to care for and empathize with others, which helps me deal with work-related issues more effectively. 9. My interactions with my family teach me patience and tolerance, which helps me perform better at work. 10. My experiences with my family help me become a more approachable and well-liked collaborator at work. 11. The positive mood I experience at home carries over to work, making me more enthusiastic and more engaged in my job. 12. To fulfill my family’s needs and aspirations, I work harder and improve my job performance. 13. My family’s understanding, trust, and support make me more confident and motivated at work. 14. My family life helps me relax both physically and mentally, preparing me for the next day’s work. |

**Individual Thriving (THR) Scale**

| **Items** | **Questionnaire items** |
| --- | --- |
| Work Thriving | 1. At work, I often learn new things. 2. Over time, I learn more and more at work. 3. I find myself continuously improving at work. 4. At work, I am not learning. 5. At work, I experience a great deal of development. 6. At work, I feel full of vitality. 7. At work, I feel energetic and spirited. 8. At work, I feel low in energy. 9. At work, I feel alert and awake. 10. At work, I look forward to each new day. |
| Family Thriving | 11. At home, I often learn new things. 12. Over time, I learn more and more at home. 13. At home, I continuously improve. 14. At home, I am not learning. 15. At home, I experience a great deal of development. 16. At home, I feel full of vitality. 17. At home, I feel energetic and spirited. 18. At home, I feel low in energy. 19. At home, I feel alert and awake. 20. At home, I look forward to each new day. |

**Employee Stewardship Behavior (ESB) Scale**

| **Items** | **Questionnaire items** |
| --- | --- |
| Employee Stewardship Behavior | 1. I have a proactive willingness to prioritize the organization’s interests over my own. 2. I demonstrate a proactive spirit that is trustworthy and commendable. 3. In my daily work, I tend to adopt a long-term orientation rather than focusing on short-term gains. |

# 3 Supplementary Material C

Supplementary Table C1 presents the hierarchical regression results for the main effect of Family-Supportive Supervisor Behavior (FSSB) on Employee Stewardship Behavior (ESB), based on composite scores and demographic controls. This analysis was conducted using SPSS to complement the latent SEM reported in the main text; the two approaches yield consistent conclusions.

Table C1. Hierarchical Regression Results for the Main Effect

| **Variable** | **ESB** | |
| --- | --- | --- |
|  | **M1** | **M2** |
| Gender | -0.049 | 0.025 |
| Age | 0.024 | 0.001 |
| Education | -0.025 | -0.013 |
| Marital Status | 0.020 | 0.000 |
| Position Level | 0.218^***^ | 0.080 |
| Supervisor Gender | 0.040 | -0.012 |
| Supervisor Age | -0.004 | 0.019 |
| FSSB | - | 0.555^***^ |
| R^2^ | 0.066 | 0.338 |
| ΔR^2^ | 0.066 | 0.272 |
| F | 4.988^***^ | 31.681^***^ |

**Note:** N=506; coefficients are standardized; ^***^p<0.001, ^**^p<0.01, ^*^p<0.05.

Supplementary Table C2 presents the hierarchical regression results for the mediating role of Work-Family Enrichment (WFE) between FSSB and ESB (Models 3–6).

Table C2. Hierarchical Regression Results for the Mediating Role of Work-Family Enrichment

| **Variable** | **WFE** | | | **ESB** | | |
| --- | --- | --- | --- | --- | --- | --- |
|  | **M3** | **M4** | **M1** | | **M5** | **M6** |
| Gender | -0.017 | 0.057 | -0.049 | | -0.040 | 0.008 |
| Age | 0.007 | -0.015 | 0.024 | | 0.020 | 0.000 |
| Education | -0.006 | 0.006 | -0.025 | | -0.022 | -0.015 |
| Marital Status | 0.048 | 0.027 | 0.020 | | -0.040 | 0.008 |
| Position Level | 0.244^***^ | 0.105^*^ | 0.218^***^ | | 0.094 | 0.048 |
| Supervisor Gender | -0.107^*^ | -0.159^***^ | 0.040 | | 0.095^*^ | 0.035 |
| Supervisor Age | -0.016 | 0.007 | -0.004 | | 0.005 | 0.017 |
| FSSB | - | 0.557^***^ | - | | - | 0.390^***^ |
| WFE | - | - | - | | 0.508^***^ | 0.298^***^ |
| R^2^ | 0.090 | 0.364 | 0.066 | | 0.301 | 0.394 |
| ΔR^2^ | 0.090 | 0.273 | 0.066 | | 0.235 | 0.093 |
| F | 7.060^***^ | 35.511^***^ | 4.988^***^ | | 26.696^***^ | 35.864^***^ |

**Note:** N=506; Standardized regression coefficients are reported; ^***^p<0.001, ^**^p<0.01, ^*^p<0.05.

Supplementary Table C3 presents the hierarchical regression results for the mediating role of Individual Thriving (THR) between FSSB and ESB (Models 7–10).

Table C3. Hierarchical Regression Results for the Mediating Role of Individual Thriving

| **Variable** | **THR** | | | **ESB** | | |
| --- | --- | --- | --- | --- | --- | --- |
|  | **M7** | **M8** | **M1** | | **M9** | **M10** |
| Gender | -0.081 | -0.006 | -0.049 | | -0.003 | 0.027 |
| Age | 0.012 | -0.011 | 0.024 | | 0.017 | 0.005 |
| Education | -0.001 | 0.011 | -0.025 | | -0.024 | -0.017 |
| Marital Status | 0.092 | 0.072 | 0.020 | | -0.032 | -0.025 |
| Position Level | 0.312^***^ | 0.171^***^ | 0.218^***^ | | 0.043 | 0.020 |
| Supervisor Gender | -0.053 | -0.106^**^ | 0.040 | | 0.070 | 0.025 |
| Supervisor Age | -0.064 | -0.041 | -0.004 | | 0.033 | 0.034 |
| FSSB | - | 0.565^***^ | - | | - | 0.357^***^ |
| THR | - | - | - | | 0.563^***^ | 0.350^***^ |
| R^2^ | 0.160 | 0.442 | 0.066 | | 0.331 | 0.406 |
| ΔR^2^ | 0.160 | 0.282 | 0.066 | | 0.266 | 0.075 |
| F | 13.506^***^ | 49.124^***^ | 4.988^***^ | | 30.801^***^ | 37.716^***^ |

**Note:** N=506. Standardized regression coefficients are reported. ^***^p<0.001, ^**^p<0.01, ^*^p<0.05.

Supplementary Table C4 presents the hierarchical regression results for the serial mediation of WFE and THR (Models 4, 11, 12, and 13).

Table C4. Hierarchical Regression Results for Serial Mediation Testing

| **Variable** | **WFE** | | **THR** | | **ESB** | |
| --- | --- | --- | --- | --- | --- | --- |
|  | **M4** | **M11** | | **M12** | | **M13** |
| Gender | 0.057 | -0.070 | | -0.032 | | 0.016 |
| Age | -0.015 | 0.008 | | -0.004 | | 0.007 |
| Education | 0.006 | 0.003 | | 0.008 | | -0.017 |
| Marital Status | 0.027 | 0.063 | | 0.060 | | -0.024 |
| Position Level | 0.105^*^ | 0.161^***^ | | 0.123^**^ | | 0.017 |
| Supervisor Gender | -0.159^***^ | 0.014 | | -0.034 | | 0.044 |
| Supervisor Age | 0.007 | -0.054 | | -0.044 | | 0.029 |
| FSSB | 0.557^***^ | - | | 0.316^***^ | | 0.308^***^ |
| WFE | - | 0.619^***^ | | 0.448^***^ | | 0.183^***^ |
| THR | - | - | | - | | 0.257^***^ |
| R^2^ | 0.364 | 0.508 | | 0.569 | | 0.423 |
| ΔR^2^ | 0.273 | 0.348 | | 0.062 | | 0.357 |
| F | 35.511^***^ | 64.123^***^ | | 72.892^***^ | | 36.241^***^ |

**Note:** N=506. Standardized regression coefficients are reported. ^***^p<0.001, ^**^p<0.01, ^*^p<0.05.

These composite-score coefficients can differ from the latent-variable estimates because they do not account for measurement error; the latent SEM estimates in the main text (Tables 4, 7, and 8) and Supplementary Table D1 serve as the primary evidence for hypothesis testing.

# 4 Supplementary Material D

Supplementary Table D1 reports the complete structural results of the hypothesized ordering model (FSSB → WFE → THR → ESB) and the reversed ordering model (FSSB → THR → WFE → ESB), estimated with the same measurement model (44 items; THR second-order) in lavaan (ML; bootstrap standard errors and bootstrap percentile 95% confidence intervals based on 5,000 resamples).

Table D1. Structural Path Estimates and Indirect Effects: Hypothesized vs. Reversed Ordering

Panel A. Hypothesized ordering (FSSB → WFE → THR → ESB)

| **Path** | **Est.** | **SE** | **95% CI** | **β** |
| --- | --- | --- | --- | --- |
| WFE ← FSSB | 0.607 | 0.071 | [0.482, 0.760] | 0.602 |
| THR ← WFE | 0.390 | 0.050 | [0.298, 0.492] | 0.517 |
| THR ← FSSB | 0.290 | 0.054 | [0.197, 0.404] | 0.381 |
| ESB ← THR | 0.660 | 0.170 | [0.361, 1.017] | 0.328 |
| ESB ← FSSB | 0.516 | 0.085 | [0.337, 0.677] | 0.337 |
| ESB ← WFE | 0.171 | 0.098 | [−0.027, 0.354] | 0.112 |
| Indirect: FSSB → WFE → ESB | 0.104 | 0.062 | [−0.017, 0.229] | — |
| Indirect: FSSB → THR → ESB | 0.191 | 0.062 | [0.091, 0.335] | — |
| Indirect: serial FSSB → WFE → THR → ESB | 0.156 | 0.044 | [0.082, 0.255] | — |
| Total indirect | 0.451 | 0.086 | [0.306, 0.644] | — |

Panel B. Reversed ordering (FSSB → THR → WFE → ESB)

| **Path** | **Est.** | **SE** | **95% CI** | **β** |
| --- | --- | --- | --- | --- |
| THR ← FSSB | 0.527 | 0.062 | [0.416, 0.656] | 0.692 |
| WFE ← THR | 0.837 | 0.128 | [0.611, 1.107] | 0.632 |
| WFE ← FSSB | 0.166 | 0.073 | [0.028, 0.316] | 0.165 |
| ESB ← WFE | 0.171 | 0.098 | [−0.027, 0.354] | 0.112 |
| ESB ← FSSB | 0.516 | 0.085 | [0.337, 0.677] | 0.337 |
| ESB ← THR | 0.660 | 0.170 | [0.361, 1.017] | 0.328 |
| Indirect: FSSB → THR → ESB | 0.348 | 0.097 | [0.181, 0.564] | — |
| Indirect: FSSB → WFE → ESB | 0.028 | 0.022 | [−0.005, 0.080] | — |
| Indirect: serial FSSB → THR → WFE → ESB | 0.075 | 0.046 | [−0.012, 0.167] | — |
| Total indirect | 0.451 | 0.086 | [0.306, 0.644] | — |

Note. N = 506. Unstandardized estimates with bootstrap standard errors; 95% confidence intervals are bootstrap percentile intervals (5,000 resamples); β = standardized estimate (z and p statistics for the standardized estimates are reported in Table 4 of the main text). Both models: χ² = 2262.385, df = 894, χ²/df = 2.531, RMSEA = 0.055, CFI = 0.898, TLI = 0.892, SRMR = 0.046; the structural part of both models is saturated, so the two orderings are equivalent reparameterizations of the same latent covariance structure and their fit is identical by construction. The difference between the two serial indirect effects was not formally tested; the comparison is descriptive.
